# Digital Therapeutic for Hwa-Byung (Korean Culture-Related Anger Syndrome) Based on Acceptance and Commitment Therapy: A Pilot Feasibility Trial

**DOI:** 10.3390/healthcare14132027

**Published:** 2026-07-07

**Authors:** Chan-Young Kwon, Boram Lee, Minjae Kim, Ji-hu Moon, Min-Gyeong Seo, Dohee Yoon

**Affiliations:** 1 Department of Oriental Neuropsychiatry, Dong-Eui University College of Korean Medicine, Busan 47227, Republic of Korea; 20256069@office.deu.ac.kr (M.K.); 20266066@office.deu.ac.kr (M.-G.S.); 2 Anti-Aging Research Center, Dong-Eui University, Busan 47227, Republic of Korea; 3 Research Institute of Korean Medicine, Dong-Eui University, Busan 47227, Republic of Korea; ideaos74@deu.ac.kr; 4 KM Science Research Division, Korea Institute of Oriental Medicine, Daejeon 34054, Republic of Korea; qhfka9357@kiom.re.kr; 5 Department of Korean Internal Medicine, Dong-Eui University College of Korean Medicine, Busan 47227, Republic of Korea; 20256071@office.deu.ac.kr

**Keywords:** Hwa-byung, digital therapeutics, acceptance and commitment therapy, pilot study, mobile health application

## Abstract

**Background/Objectives**: Hwa-byung (HB) is a culture-bound syndrome characterized by prolonged suppression of anger and somatic complaints. No evidence-based digital therapeutic (DTx) has been developed for HB. We evaluated the feasibility, user experience (UX), and preliminary clinical effects of an acceptance and commitment therapy-based DTx, Hwa-free, for HB. **Methods**: Adults aged 19–80 years diagnosed with HB were enrolled in a four-week app-based intervention with assessments at Weeks 0, 2, 4, and 8. The primary outcome was UX via a 22-item survey at Week 4. Secondary outcomes included HB scales, depression, anxiety, anger, psychological flexibility, and health-related quality of life. **Results**: Of 45 screened, 30 were enrolled; 28 constituted the modified intention-to-treat population. Mean app use was 20.3 ± 7.8 days (72.6% adherence). All adverse events were assessed as probably or definitely unrelated to the intervention. UX positive response rates exceeded 80% across video content (82.8–89.7%), HB self-assessment (86.2%), meditation (86.2%), and in-app guidance (85.7%). Within-group changes at Week 4 were observed in 11 of 18 clinical scales, including HB Symptom Scale (Δ = −9.8, d = −0.92), Beck Depression Inventory-II (Δ = −13.3, d = −1.11), and state anger (Δ = −7.8, d = −0.96); these are uncontrolled pre–post changes not attributable to the intervention. The HB screening-positive rate declined from 100% at baseline to 55.6% at Week 8. **Conclusions**: Hwa-free demonstrated adequate feasibility, acceptable UX, and preliminary within-group symptom changes. A fully powered randomized controlled trial is warranted.

## 1. Introduction

Hwa-byung (HB), literally meaning anger illness, is a Korean culture-bound syndrome first introduced into the psychiatric literature in 1983 [1]. It is characterized by the prolonged suppression of anger and resentment, manifesting as a heterogeneous cluster of somatic and psychological symptoms, including chest tightness, a rising or burning sensation, heat in the face or chest, a lump-like sensation in the throat or epigastrium, palpitations, and a deep-seated feeling of injustice (*han*) [2]. The condition has been acknowledged by the American Psychiatric Association as a cultural concept of distress [3], and is estimated to affect between 4.2% and 13.3% of the Korean adult population, disproportionately affecting middle-aged women with limited social support [4]. However, recently, a high prevalence rate of 36.3% has been reported among the Korean MZ generation, suggesting that this disease persists in modern Korean culture [5]. This wide prevalence range reflects methodological heterogeneity across studies, including differences in target populations (middle-aged community samples vs. younger generations) and diagnostic instruments (clinician-administered diagnostic tool vs. self-report screening scales).

HB is phenomenologically distinct from depression and anxiety disorders in three key respects. First, its core affective content—suppressed injustice (*han*), resentment (*bun*), and indignation (*eok-ul-ham*)—is qualitatively different from the sadness and hopelessness characteristic of depression or the fear and apprehension of anxiety [2]. Second, HB is associated with prominent culture-specific somatic manifestations, particularly chest tightness, a rising heat sensation, and a lump-like sensation in the throat or epigastrium, which represent culturally mediated channels for suppressed affect rather than non-specific somatic symptoms of mood or anxiety disorders [1,2]. Third, HB patients characteristically attribute their distress to interpersonal injustice and social oppression—a cognitive-attributional pattern not targeted by existing digital interventions for depression or anxiety. These distinctions necessitate a culturally tailored therapeutic approach that directly addresses the suppression-based pathomechanism and validates the culture-specific emotional experience of HB.

Current evidence-based treatments for HB include pharmacotherapy, psychotherapy, and Korean medicine (KM) interventions, but access to specialist care remains constrained by geographic, economic, and stigma-related barriers [4,5,6]. Digital therapeutics (DTx), prescription or over-the-counter software delivering evidence-based therapeutic content, represent a scalable solution to these access challenges [7], and smartphone-based interventions have demonstrated efficacy for depression, anxiety, and insomnia in randomized trials [8,9]. Nevertheless, the digital mental health evidence base remains mixed; engagement decay, high dropout rates, and modest effect sizes relative to face-to-face treatment are consistent findings in the literature [8]. However, no DTx has been developed or rigorously evaluated specifically for HB.

Acceptance and commitment therapy (ACT) is a contextual cognitive-behavioral approach that targets experiential avoidance and promotes psychological flexibility through acceptance, defusion, values clarification, and committed action [10]. ACT has demonstrated efficacy across a range of emotional and somatic conditions [11], and its theoretical framework aligns well with the psychopathology of HB, in which chronic suppression of anger represents a form of experiential avoidance [2,4]. The KM Clinical Practice Guideline for HB recommends ACT as a first-line psychotherapeutic approach (Grade A evidence) [4]. ACT is particularly well-suited to HB because its core construct of experiential avoidance directly maps onto the suppression-based HB pathomechanism; unlike cognitive-behavioral therapy, which targets cognitive distortions, ACT works through acceptance and defusion [10]—mechanisms more appropriate for a condition rooted in suppressed legitimate grievances rather than irrational cognitions. Mindfulness-based interventions, while sharing conceptual overlap, lack the values-clarification and committed action components that may be necessary to address the identity-level suffering and long-term emotional constriction characteristic of HB.

Hwa-free(version 1.1.6; DYPHI Inc., Daejeon, Republic of Korea) is an ACT-based DTx application developed specifically for individuals with HB, integrating structured psychoeducational content, behavioral skills training (diaphragmatic breathing, relaxation, meditation), and daily self-monitoring [12]. Each module was designed to target a distinct component of the ACT hexaflex and the HB pathomechanism: weekly psychoeducational video content promotes psychoeducation and committed action by framing HB within an ACT model of emotional suppression; diaphragmatic breathing training targets autonomic dysregulation and provides a physiological anchor for present-moment awareness; relaxation therapy and meditation exercises cultivate defusion and acceptance of aversive internal states; and the three-line journaling module supports values clarification and cognitive defusion through structured daily reflection. However, the scalability advantage of DTx may be limited among older adults and those with limited smartphone familiarity; digital literacy, technology comfort, and sustained engagement represent important implementation barriers that must be considered when targeting populations such as HB patients, who are disproportionately middle-aged or older [4,7].

The present study aimed to (1) evaluate the feasibility and adherence of Hwa-free over a four-week intervention period; (2) assess user experience (UX) as the primary outcome; and (3) generate preliminary data on clinical effect across HB-related psychological and physiological outcomes to inform the design of a subsequent randomized controlled trial (RCT).

## 2. Materials and Methods

### 2.1. Study Design and Setting

A single-arm, single-center pilot study was conducted at the Korean Medicine Hospital of Dong-eui University, Busan, Republic of Korea. The study was approved by the Institutional Review Board of Dong-eui University Korean Medicine Hospital (DH-2025-15; 15 October 2025) and submitted to the Clinical Research Information Service (CRIS; KCT0011105) on 21 October 2025, prior to the first participant’s enrolment on 30 October 2025. All participants provided written informed consent prior to enrolment. A detailed study protocol has been published elsewhere [12]. Reporting follows the CONSORT extension for pilot and feasibility trials, as applicable to single-arm designs [13].

### 2.2. Participant Recruitment and Eligibility

Eligible participants were adults aged 19–80 years who met diagnostic criteria for HB as confirmed by the Hwa-byung Diagnostic Interview Schedule (HBDIS) [14]. Key exclusion criteria included initiation or substantial modification of any treatment for HB within the preceding four weeks; concurrent participation in an interventional clinical trial; planned initiation of new HB treatment during the study period; diagnosis of uncontrolled schizophrenia, bipolar disorder, or other serious psychiatric disorders within the preceding six months with ongoing active treatment; severe chronic or terminal illness; or inability to comply with study procedures as judged by the investigator. Participants were permitted to continue pre-existing stable treatments throughout the study period; however, initiation of new treatments for HB during the study was not permitted. Participants were recruited via in-hospital posters from 30 October 2025 to 22 January 2026. Target sample size was 30 participants, determined pragmatically consistent with the convention for single-arm pilot feasibility trials [15], with a 20% attrition allowance. Formal power analysis is not applicable to this pilot feasibility trial, the primary purpose of which was estimation of feasibility parameters and effect sizes to inform the sample size calculation for the planned multicenter confirmatory RCT.

### 2.3. Intervention

**Figure 1:**
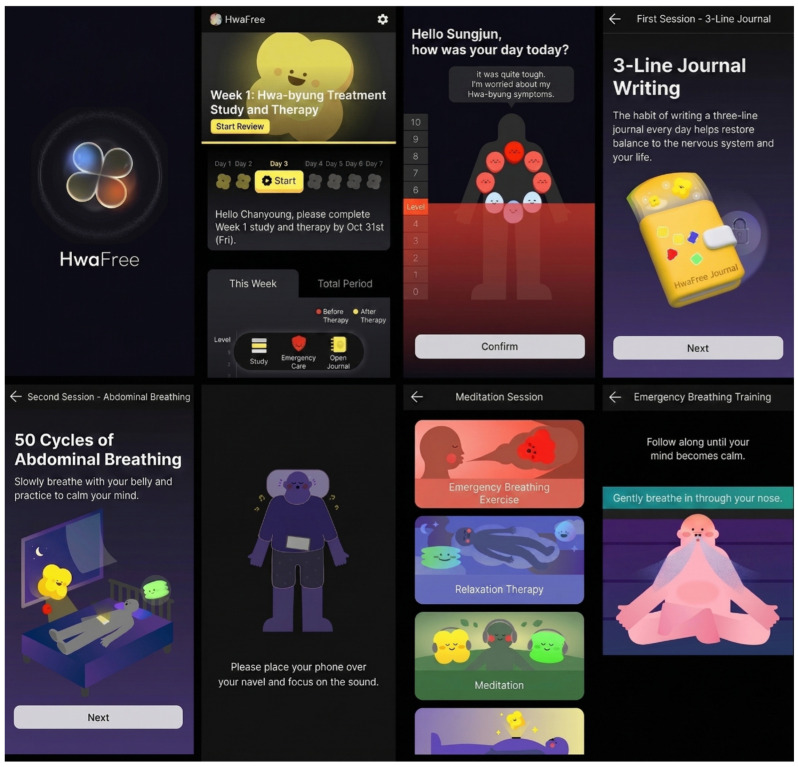
Screenshots of Hwa-free smartphone application interface. **Note**. The screenshots illustrate the comprehensive digital therapeutic components for Hwa-byung management, including the home interface and treatment progress tracking; daily symptom and stress level assessment; a three-line therapeutic journal; guided abdominal breathing sessions utilizing smartphone-based biofeedback; and emergency relief modules featuring relaxation therapy and guided meditation.

Hwa-free is a smartphone application delivering a structured four-week ACT-based program (Figure 1). A full description of the intervention modules and their theoretical basis is provided in the published protocol [12], briefly, the program comprises weekly ACT-informed psychoeducational video content, daily diaphragmatic breathing training (with smartphone accelerometry for breath detection), relaxation therapy, meditation exercises, a three-line journaling module, and a HB symptom self-assessment feature. Automated push notifications were delivered at 22:00 daily to encourage engagement. The intervention period was four weeks (28 days); app access was programatically restricted after Week 4, and participants had no access to the application during the subsequent four-week observation period (Weeks 4–8).

### 2.4. Outcome Measures

#### 2.4.1. Feasibility and Adherence Measures

Feasibility was operationalized as daily app use over the 28-day intervention, recorded at two engagement levels: (1) minimum use—defined as app launch with access to at least one functional feature (reflecting any app engagement); and (2) full session completion—defined as completion of the mandatory diaphragmatic breathing module (reflecting meaningful therapeutic exposure). Both metrics are reported to provide a transparent picture of the dose of engagement.

#### 2.4.2. Primary Outcome

The primary outcome was UX at Week 4, assessed by a self-developed 22-item survey administered as a self-completed paper questionnaire at the Week 4 clinical visit. The survey was developed iteratively: an initial item pool was generated based on the app’s functional domains, reviewed for content validity by two clinical experts in HB and digital health, and refined following a pilot administration to five individuals with HB experience, who completed the questionnaire and provided feedback on unclear or problematic items. Items 1–17 and 20–22 used a 0–4 Likert scale (0 = not at all, 4 = strongly agree); item 16 (barriers to diary use) was reverse-scored. Items 18 and 19 assessed usage frequency (ordinal, 4 categories) and reasons for non-daily use (categorical, multiple-response), respectively; these items were analyzed separately and are not included in the domain scoring. Items were grouped into seven a-priori domains: Video Content, Diaphragmatic Breathing, Relaxation Therapy, Meditation Therapy, Three-line Diary, Self-Assessment, and App Usability. Positive response was defined as a score ≥ 3 (or ≤1 for item 16). Internal consistency was assessed by Cronbach’s α.

#### 2.4.3. Secondary Outcomes

Secondary outcomes included the HB scale including HB Symptom Scale (HBSS; 0–60) and HB Personality Scale (HBPS; 0–64) [16], Beck Depression Inventory-II (BDI-II) [17], State-Trait Anxiety Inventory (STAI; state, STAI-S; trait, STAI-T) [18], State-Trait Anger Expression Inventory (STAXI; state anger, STAXI-S; trait anger, STAXI-T; anger control; anger-out; anger-in) [19], and a 15-item visual analogue scale (VAS; 0–100 mm) for individual HB symptoms. Additional measures included the Acceptance and Action Questionnaire-II (AAQ-II; 8 items, 1–7 Likert scale; higher scores indicate greater experiential avoidance) [20], EQ-5D-5L (Korean utility weights) [21], and heart rate variability (HRV; Canopy9 Plus; IEMBIO Co., Ltd., Chuncheon, Republic of Korea; 1-min measurement following 5 min of seated rest) [22]. HB screening positivity was defined as HBSS ≥ 30 [16]. In the absence of an established MCID or validated responder threshold for the HBSS, a ≥30% decrease from baseline was adopted as an exploratory responder criterion for descriptive purposes only; this threshold was not pre-specified and findings should be interpreted as hypothesis-generating. Clinical assessments were conducted at Weeks 0, 2, 4, and 8 (or Weeks 0, 4, and 8 for AAQ-II and EQ-5D-5L; Weeks 0 and 4 for HRV).

#### 2.4.4. Safety Measures

Adverse events (AEs) were assessed at Weeks 2 and 4. Investigators recorded the occurrence of AEs and assessed their causal relationship to the intervention using a 6-point causality scale (1 = definitely related; 2 = probably related; 3 = possibly related; 4 = probably not related; 5 = definitely not related; 6 = unknown). MedDRA coding was not applied in this pilot study, which represents a limitation relative to regulatory-grade clinical trial reporting standards.

### 2.5. Statistical Analysis

Two analysis populations were defined: (1) the modified intention-to-treat (mITT) population (primary analysis; ≥1 day of app use and ≥1 post-baseline assessment) and (2) the per-protocol (PP) population (sensitivity analysis; Week 4 assessment completed and ≥14 use days). Missing data were handled by complete case analysis for each pairwise comparison; participants with missing data at a given timepoint were excluded only from analyses involving that timepoint. As a sensitivity analysis pre-specified in the published protocol [12], last observation carried forward (LOCF) was applied to the seven primary clinical scales (HBSS, BDI-II, STAI-S/T, STAXI-S/T, AAQ-II) for the primary Week 0-to-Week 4 comparison in the mITT population. Normality was evaluated by the Shapiro–Wilk test. Pre–post comparisons used paired *t*-tests (normally distributed difference) or Wilcoxon signed-rank tests (non-normal), with Cohen’s d or r as effect size, respectively. Multiple comparisons were corrected using the Benjamini–Hochberg false discovery rate (FDR), applied separately within each comparison set (Week 0 vs. Week 4; Week 0 vs. Week 8; Week 4 vs. Week 8). Given the small sample size, all analyses should be interpreted as descriptive and exploratory; inferential statistics are reported to generate hypotheses for the confirmatory RCT rather than to establish efficacy. Repeated-measures changes were assessed by Friedman’s test with Dunn’s post hoc test (Bonferroni-corrected) as the primary repeated-measures analysis. Mixed models for repeated measures (MMRMs; random intercept, baseline as covariate, restricted maximum likelihood) were additionally fitted as a supplementary confirmatory analysis. Adherence–outcome associations used Spearman’s ρ. An exploratory mediation analysis examined whether change in AAQ-II (ΔAAQ-II) mediated HBSS change (ΔHBSS), using ordinary least squares regression and bootstrap resampling (*N* = 5000; 95% CI); results are hypothesis-generating only given the single-arm design. All analyses were conducted in Python (version 3.12; Python Software Foundation, Wilmington, DE, USA) using pandas (version 3.0.4), scipy (version 1.18.0), statsmodels (version 0.14.6), and scikit-posthocs (version 0.14.0). Significance threshold: α = 0.05, two-tailed.

## 3. Results

### 3.1. Participant Characteristics

**Figure 2:**
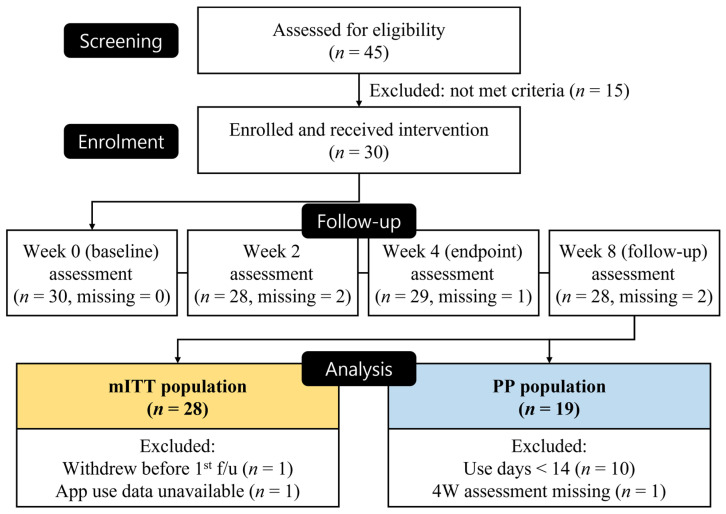
Flow diagram of participant enrolment and follow-up. **Abbreviations**: mITT, modified intention-to-treat; PP, per-protocol. **Note**. All screen failures were attributable to non-fulfilment of HB diagnostic criteria on the HBDIS (*n* = 15).

Of 45 individuals screened for eligibility, 15 were excluded for not meeting the HB diagnostic criteria on the HBDIS and 30 were enrolled (Figure 2). The first participant was enrolled on 30 October 2025; the final study assessment was completed on 31 March 2026. Of the 30 enrolled participants, 15 (50.0%) reported concurrent medications or treatments at the time of enrolment, predominantly for pre-existing stable chronic conditions including cardiovascular disease (hypertension, dyslipidaemia; *n* = 7), metabolic disorders (diabetes mellitus, hypothyroidism, osteoporosis; *n* = 5), and menopausal symptoms (hormonal therapy; *n* = 1); one participant (3.3%) was concurrently taking a low-dose hypnotic agent (triazolam 0.25 mg) for insomnia, and two participants used herbal formulas during the study period. No participant was receiving concurrent psychotherapy. All concurrent medications had been stable for ≥4 weeks prior to enrolment per inclusion criteria (Table S1).

Two enrolled participants were excluded from the mITT population: one who withdrew before the Week 2 visit citing a medical reason that precluded smartphone use (no post-baseline clinical assessments available), and one whose app use data were unavailable (due to private VPN settings) and conservatively treated as zero days (mITT: *n* = 28) (Table S2). Nineteen participants met per-protocol criteria (PP: *n* = 19). Participants were predominantly female (86.7%; *n* = 26/30), with a median age of 55.5 years (IQR 48.2–65.0) and a median HB duration of 8.5 years (IQR 4.2–20.8). Mean BMI was 24.5 ± 3.0 kg/m^2^. At baseline, all mITT participants (100%) were screen-positive for HB (HBSS ≥ 30; mean HBSS: 46.9 ± 7.8). Mean BDI-II was 38.4 ± 10.4 and mean AAQ-II was 39.7 ± 9.6 (Table 1).

### 3.2. Feasibility and Adherence Outcomes

Participants used the app on a mean of 20.3 ± 7.8 days out of 28 (minimum use-day adherence: 72.6% ± 27.8%; mITT, *n* = 28). The mean number of days on which the complete diaphragmatic breathing session was performed was 11.9 ± 9.5 (full session completion rate: 42.5% ± 33.8%). Mean daily usage time was 26.9 ± 18.3 min (cumulative total: 536.3 ± 470.9 min). ACT mandatory session completion averaged 10.0 ± 2.0 out of a maximum of 12 sessions (83.3% completion rate; 12 participants [42.9%] completed all 12 sessions). Module-level engagement showed variability: the diaphragmatic breathing module was used on a mean of 12.4 ± 9.7 occasions compared with 5.1 ± 9.8 occasions for the relaxation therapy/meditation module. UX positive response rates were 82.8–89.7% for video content, 86.2% for meditation therapy, and 86.2% for relaxation therapy. Recruitment yield was 66.7% (30 of 45 screened). Detailed engagement metrics are presented in Table S3.

### 3.3. Safety Outcomes

One participant withdrew from the study before the Week 2 visit due to a personal ophthalmic condition that precluded smartphone use, unrelated to the intervention (dropout rate: 3.3%); this participant had no post-baseline clinical assessments and was excluded from the mITT population. A total of eight non-serious AEs were reported across the intervention period: three events in two participants (6.7%) during Weeks 0–2 (insomnia onset, *n* = 1; chronic tonsillitis and pre-hypertension, *n* = 1), and five events in three participants (10.0%) during Weeks 2–4 (hypertension and reflux esophagitis, *n* = 1; acute nasopharyngitis and allergic dry eye syndrome, *n* = 1; generalized myalgia, *n* = 1). All AEs were assessed by the investigator as probably or definitely unrelated to the study intervention, and no serious AEs were reported (Table S4).

### 3.4. Primary Outcome: User Experience

Positive response rates (score ≥ 3) were highest for Week 4 video content (item 4: 89.7%), the HB self-assessment function (item 17: 86.2%), meditation therapy effectiveness (item 9: 86.2%), in-app guidance clarity (item 20: 85.7%), and Week 2 video content (item 2: 82.8%). The lowest-rated items were diaphragmatic breathing breath-count accuracy (item 12: 41.4%), ease of placing the phone on the abdomen during breathing (item 11: 48.3%), and ease of following breathing instructions (item 13: 62.1%). The overall internal consistency of the UX questionnaire was Cronbach’s α = 0.9067. Domain-level internal consistency ranged from α = 0.69 (Three-line Diary, the lowest) to α = 0.94 (Relaxation Therapy); item-level score distributions are provided in Table S5. Daily app use was reported as daily by 44.8% and 4–5 times per week by 31.0% of respondents (Table 2; Figure S1).

### 3.5. Secondary Outcomes: Clinical Scale Changes (Week 4, mITT)

**Figure 3:**
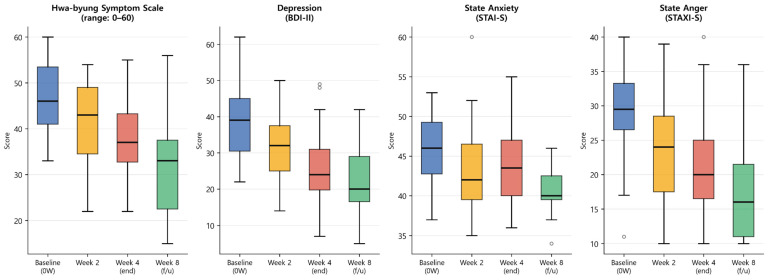
Changes in state/acute clinical outcomes across four timepoints in the mITT population. **Abbreviations**: BDI-II, Beck Depression Inventory-II; STAI-S, State-Trait Anxiety Inventory state subscale; STAXI-S, State-Trait Anger Expression Inventory state anger subscale. **Note**. Box plots show median, interquartile range, and individual paired trajectories (Week 0 to Week 4). Colors represent timepoints: baseline (Week 0, blue), Week 2 (orange), Week 4 (red), Week 8 follow-up (green). This figure presents state and acute symptom measures (Hwa-byung Symptom Scale, Depression [BDI-II], State Anxiety [STAI-S], and State Anger [STAXI-S]); trait- and disposition-level scales (HBPS, STAI-T, STAXI-T, AAQ-II, and others) are presented in Table 3. Of 18 clinical scales analyzed, 11 reached statistical significance after FDR correction at Week 4. The largest within-group changes were observed for BDI-II [Δ = −13.3 (95% CI −17.9 to −8.6), 34.2% reduction, Cohen’s d = −1.11, *p* < 0.001], STAXI-S [Δ = −7.8 (95% CI −11.0 to −4.7), 27.1% reduction, d = −0.96, *p* < 0.001], and HBSS [Δ = −9.8 (95% CI −14.0 to −5.7), 20.9% reduction, d = −0.92, *p* < 0.001]. Statistically significant changes were also found for HBPS [Δ = −9.1 (95% CI −13.6 to −4.7), d = −0.79], STAI-T [Δ = −4.8 (95% CI −7.0 to −2.7), d = −0.87], STAXI-T [Δ = −6.6 (95% CI −9.6 to −3.5), d = −0.83], AAQ-II [Δ = −7.0 (95% CI −10.5 to −3.5), d = −0.78], Anger-Out [Δ = −3.1 (95% CI −4.9 to −1.4), d = −0.71], Anger-In [Δ = −3.9 (95% CI −6.1 to −1.7), d = −0.70], EQ-5D-5L VAS [Δ = +13.6 (95% CI +6.1 to +21.0), d = 0.71], and EQ-5D-5L Index [Δ = +0.083 (95% CI +0.024 to +0.142), r = 0.54, Wilcoxon]. Fourteen of 15 HB-VAS items showed statistically significant improvement; item 10 (dry mouth) did not reach significance (Table S7). HRV indices showed no significant change (all FDR-adjusted *p* > 0.05; Figure S2). PP sensitivity analyses yielded directionally consistent results with comparable effect magnitudes across most significant scales; however, HB-VAS improvements were substantially attenuated, with only one item (accumulated anger/rage) reaching significance after FDR correction, likely reflecting reduced statistical power in the smaller PP sample (*n* = 19). LOCF sensitivity analysis for the seven primary clinical scales yielded results identical to the complete-case analysis: no Week 4 data were missing within the mITT population (zero values imputed; all directional findings consistent with complete-case results) (Table S8).

Pre–post comparisons from baseline to Week 4 in the mITT population (*n* = 28) are summarized in Table 3 and Figure 3. Preliminary within-group changes were already apparent at Week 2 for several state measures: HBSS declined from 47.1 ± 7.8 at baseline to 41.2 ± 8.6 at Week 2, BDI-II from 38.9 ± 10.4 to 30.6 ± 8.6, STAXI-S from 28.9 ± 7.2 to 24.0 ± 7.9, and STAI-S from 45.0 ± 4.8 to 42.7 ± 5.0. Repeated measures post hoc analysis (Dunn’s test, Bonferroni-corrected) indicated that HBSS and BDI-II did not reach significance at the Week 2 timepoint (*p* = 0.187 and *p* = 0.055, respectively). Detailed post hoc results for trait-level scales across all timepoints are presented in Table S6.

### 3.6. Follow-Up Outcomes (Week 8, mITT)

Outcomes at Week 8 are presented in Table S9. The HB screening-positive rate declined from 100% at baseline to 82.1% at Week 4 and 55.6% at Week 8 (Table S10). All 15 HB-VAS items showed significant improvement from baseline to Week 8. Friedman’s test identified significant repeated-measures effects in 27 of 28 scales (Table S6). Post hoc analysis (Dunn’s test, Bonferroni-corrected) indicated that improvements in HBSS and BDI-II were primarily driven by the Week 0 versus Week 4 and Week 0 versus Week 8 contrasts; the Week 2 versus Week 4 and Week 4 versus Week 8 contrasts were not significant for either scale, suggesting that change was established by Week 4 and maintained through the app-free observation period. STAI-S reached significance only at the Week 0 versus Week 8 contrast (*p* = 0.0015). Notably, for AAQ-II, the Week 4 versus Week 8 contrast was also significant (*p* = 0.041), indicating that psychological flexibility continued to improve during the app-free observation period. Detailed post hoc comparisons for all scales are presented in Table S6. MMRM confirmed time-related effects in all six primary scales, with all models converging (Table S11). As an exploratory observation, the proportion of participants showing a ≥30% decrease in HBSS from baseline was 28.6% (8/28) at Week 4 and 59.3% (16/27) at Week 8 (Table S12).

### 3.7. Exploratory Analyses

Results of the exploratory association analysis examining concurrent changes in psychological flexibility and HB symptoms are summarized in Table S13 and Figure S3. ΔAAQ-II was significantly correlated with ΔHBSS (Spearman ρ = 0.53, *p* = 0.004), and the bootstrap indirect estimate excluded zero (a × b = −3.02, 95% CI −6.42 to −0.40; R^2^ = 0.46). Adherence (minimum and maximum use days) was not significantly correlated with clinical improvement in any outcome (Spearman’s ρ, all FDR-adjusted *p* > 0.05; Table S14).

## 4. Discussion

The DTx for HB demonstrated adequate feasibility: mean adherence of 72.6% (mITT, *n* = 28) over the 28-day intervention exceeded the upper bound of engagement rates typically reported for mental health smartphone applications in clinical trials (40–70%) [23,24], and no participant withdrew due to AEs related to the intervention. Regarding recruitment feasibility, all screen failures were attributable exclusively to HBDIS non-fulfilment (*n* = 15, 33.3%), suggesting that in-hospital poster recruitment effectively targeted a symptomatic population; future trials may benefit from supplementing hospital-based recruitment with community-based and online recruitment strategies to improve screening yield and reduce self-selection bias. UX was rated favorably across most functional domains, with positive response rates exceeding 72% for video content, relaxation, and meditation modules, supporting the acceptability of these core therapeutic components. UX feedback identified the diaphragmatic breathing module as the primary target for technical refinement: breath-count accuracy (41.4% positive) and the requirement to place the phone on the abdomen (48.3% positive) were the lowest-rated feature. Improving the accuracy of the motion-sensing algorithm used for breath-count detection should be prioritized in future iterations. Among participants who did not use the app daily (*n* = 21), the most commonly reported barrier was insufficient reminders or alerts (42.9%), followed by content perceived as too long or burdensome (23.8%); only 9.5% cited low perceived immediate need. These findings suggest that optimizing notification frequency and timing, and reducing session length or perceived burden, should be prioritized in subsequent iterations.

For contextualization, the KM Clinical Practice Guideline for HB reports a meta-analytic between-group mean difference of −13.65 points on the HBSS (95% CI −18.24 to −9.05) for face-to-face group ACT versus wait-list control [4]. The within-group HBSS change observed in the present study (Δ = −9.8) is directionally consistent with this estimate, although direct comparison is confounded by the absence of a control condition and differences in delivery format and session structure. These findings are directionally consistent with ACT-based face-to-face outcomes [25]; however, within-group effect sizes are structurally inflated relative to between-group estimates in the absence of a control condition, as they incorporate spontaneous remission, regression to the mean, and expectancy effects. Accordingly, the apparent similarity in magnitude between the within-group effect observed here and the meta-analytic between-group estimate cannot be interpreted as evidence of equivalent or superior efficacy. This hypothesis warrants confirmation in a head-to-head RCT.

The pattern of within-group change is theoretically coherent within the ACT framework: ACT targets experiential avoidance and promotes acceptance of aversive internal states [10], mechanisms directly relevant to the anger suppression that characterizes HB [2,4]. AAQ-II showed significant pre–post change (d = −0.78), and the exploratory association analysis indicated a significant co-occurring relationship between ΔAAQ-II and ΔHBSS (indirect effect estimate −3.02, 95% CI −6.42 to −0.40). As both variables were measured concurrently as pre–post differences over the same interval, this finding represents correlated change consistent with ACT theory rather than evidence of mediation. Of particular interest, significant change was also observed in HBPS (d = −0.79), suggesting that the intervention may have engaged dispositional vulnerability factors, habitual emotional suppression and experiential avoidance [16], beyond acute symptom modulation; this hypothesis warrants investigation in future controlled studies with repeated trait-level assessments.

During the four-week app-free observation period (Weeks 4–8), improvement continued across most outcomes: AAQ-II showed a significant further increase between Weeks 4 and 8 (post hoc *p* = 0.041), and the exploratory HBSS responder rate doubled from 28.6% at Week 4 to 59.3% at Week 8. This pattern is consistent with the hypothesis that participants may have internalized ACT-based coping skills acquired during the active program; however, this interpretation is speculative in the absence of a control group, and alternative explanations including natural recovery trajectory and continued uncontrolled environmental factors cannot be excluded. This finding is consistent with delayed skill consolidation reported for ACT interventions in other emotional disorders [10], but requires confirmation in a controlled design. Similar sustained effects have been reported in smartphone-delivered ACTs. In a study by Gentili et al., an 8-week smartphone-delivered ACT intervention was applied to patients with chronic pain, and the effects on psychological flexibility, depression, and anxiety were found to have lasted for more than 6 months [26]. In the pathology of HB, the failure to accept or avoid stressful situations is emphasized as a key cause of HB development [2,4]. Therefore, while it is possible that ACT acts on this etiology to contribute to the maintenance of long-term effects, further research is required to clarify the underlying mechanism.

State anxiety (STAI-S) and anger control did not reach statistical significance at Week 4. The STAI-S change at Week 4 (Δ = −1.6) fell substantially below the published anchor-based MCID of approximately 10 points [27], though STAI-S did reach significance at the Week 0 versus Week 8 contrast (*p* = 0.0015), suggesting a possible delayed response. The absence of improvement in anger control is conceptually consistent with the ACT model, which emphasizes acceptance over deliberate control of aversive emotions [10]; accordingly, anger control was not a primary therapeutic target of the intervention. The absence of significant HRV change likely reflects both the adoption of a one-minute measurement epoch, limiting reliability relative to the five-minute standard [22], and insufficient engagement with the diaphragmatic breathing module, which may be necessary for meaningful autonomic restoration [28]; autonomic nervous system adaptation may also require longer intervention periods than four weeks. The non-significant change in state anxiety (STAI-S) at Week 4 may reflect the situational nature of the construct—STAI-S captures momentary anxiety at the time of assessment, which may be less sensitive to a four-week app-based intervention than trait-level measures; By contrast, trait anxiety (STAI-T) improved significantly at Week 4 (d = −0.87), suggesting that the intervention may have preferentially influenced dispositional emotional regulation over acute state responses.

Adherence was not significantly associated with clinical improvement across any outcome. Two explanations merit equal consideration: (1) a threshold effect, whereby a minimum level of engagement is necessary but additional exposure above that threshold yields diminishing marginal returns—consistent with the observation that mean adherence (72.6%, mITT) exceeded the commonly cited 70% benchmark; or (2) insufficient statistical power in this small sample (*n* = 28) to detect a dose–response relationship if one exists. These two explanations cannot be distinguished in the current design, and a sufficiently powered RCT with pre-specified adherence–outcome analyses is required to resolve this question. Of note, 8 of 28 mITT participants (28.6%) used the app on fewer than half the available days (fewer than 14 of 28 days), indicating that engagement was uneven across the sample. Beyond the trial setting, deploying this DTx at scale raises practical questions that the present pilot does not resolve. The current study was conducted under direct clinical supervision; outside this context, the role of KM practitioners in monitoring engagement and managing AEs would need to be defined. Regarding healthcare system integration, DTx in Korea follow a defined regulatory pathway: following the confirmatory RCT, the intervention would require new health technology assessment by the National Evidence-based Healthcare Collaborating Agency, followed by a coverage determination by the Health Insurance Review and Assessment Service before reimbursement within the national health system could be established [29]. No reimbursement precedent yet exists for DTx within KM in Korea, and establishing their health economic value will depend on cost-effectiveness data from the confirmatory RCT.

Several limitations warrant acknowledgement. First, the absence of a control condition precludes any causal attribution of the observed within-group changes to the intervention itself; regression to the mean, Hawthorne effects [30], expectancy effects, and natural remission cannot be excluded. Second, no a priori feasibility thresholds or progression criteria were defined. For descriptive reference, the observed findings were evaluated against benchmarks commonly cited in the digital mental health feasibility literature: adherence ≥ 70% (minimum use-day rate; this trial: 72.6%) [23,24] and recruitment yield ≥ 50% of screened individuals (this trial: 66.7%, 30/45); UX acceptability was assessed descriptively from observed positive response rates across content domains. These benchmarks, along with explicit UX acceptability criteria, will be prospectively pre-specified as progression criteria in the protocol of the planned confirmatory RCT. Third, generalizability is restricted by the cultural specificity of HB [1], the predominantly female sample (86.7%), and single-center recruitment from a KM hospital; findings may not be representative of male patients, community populations, or individuals without prior engagement with KM services. With respect to cross-cultural transferability, the core ACT-based mechanisms—acceptance, defusion, values clarification, and committed action—are transdiagnostic and potentially applicable to populations characterized by chronic emotional suppression, whereas the HB-specific psychoeducational framing (e.g., Confucian gender role expectations) is uniquely tied to Korean cultural contexts [1,2] and would require adaptation for other populations. More broadly, chronic emotional suppression driven by structural injustice—including racial discrimination—may represent a common etiological pathway across cultures [31], and cross-cultural validation of this intervention framework is warranted. Self-selection bias is also likely, as recruitment via in-hospital posters may have favored highly motivated individuals already interested in KM-based or digital health interventions. Fourth, the UX survey is a study-specific instrument whose psychometric properties have not been fully established: construct validity, criterion validity, test–retest reliability, and factor structure were not assessed, and direct comparison with standardized instruments such as the System Usability Scale [32] was not possible, which together limit the interpretability of UX as the primary outcome. The Three-line Diary domain showed the lowest internal consistency (α = 0.69), and may warrant item revision in future iterations. Accordingly, UX findings from this study should be interpreted with considerable caution until the instrument undergoes more extensive psychometric validation. Fifth, LOCF sensitivity analysis confirmed that missing data did not bias the primary endpoint findings; however, the potential influence of missing data at follow-up (6.7% at Week 8) on secondary outcomes cannot be fully excluded. Sixth, reliance on self-reported outcomes in an unblinded design introduces the possibility of response bias, social desirability bias, and expectancy effects. Seventh, digital literacy and technology familiarity were not assessed, limiting our ability to evaluate whether engagement patterns or UX ratings were influenced by prior smartphone experience. Eighth, HRV was measured using a 1-min recording (Canopy9 Plus), substantially less reliable than the standard 5-min protocol [22]. The shorter recording duration was a pragmatic constraint of the clinical pilot setting, in which 5-min measurement equipment was unavailable; this trade-off between measurement feasibility and reliability likely reduced sensitivity and may have contributed to the absence of statistically significant HRV findings. Ninth, the 4-week intervention period was deliberately chosen to maximize feasibility and adherence in a pilot context, but may be insufficient to fully consolidate ACT-based skills; whether a longer intervention period yields additional benefit, without compromising the adherence advantage observed here, should be examined in future trials. The 8-week observation period is similarly too brief to assess long-term maintenance beyond this window. Finally, HB-VAS findings should be interpreted with caution given substantial attenuation in the PP sensitivity analysis. In addition, the exploratory analysis using the ≥30% HBSS reduction criterion was introduced post hoc. Because a validated responder threshold or MCID for the HBSS has not yet been established, these proportions should be interpreted strictly as hypothesis-generating descriptive data rather than definitive evidence of clinical efficacy.

## 5. Conclusions

Hwa-free demonstrated adequate feasibility and acceptable user experience in this single-arm pilot trial. Preliminary within-group changes were observed across HB-related psychological outcomes, with effects sustained through a four-week app-free observation period; however, these findings cannot be attributed to the intervention in the absence of a control condition. The observed effect sizes and feasibility parameters provide the foundation for a fully powered multicenter RCT incorporating an active or waitlist control condition, which is required to establish the efficacy of this ACT-based DTx for HB.

## Figures and Tables

**Table 1:** Baseline characteristics of enrolled participants.

Characteristics	Value (*N* = 30)
Age (years) ^†^	55.5 (48.2–65.0)
Female, *n* (%)	26 (86.7)
BMI (kg/m^2^)	24.48 ± 2.97
HB duration (years)	8.5 (4.2–20.8)
Psychiatric history, *n* (%)	3 (10.0)
Other medical history, *n* (%)	25 (83.3)
HBPS	47.70 ± 9.63
HBSS	46.93 ± 7.83
BDI-II	38.37 ± 10.40
STAI-S	44.97 ± 4.81
STAI-T	47.90 ± 5.50
STAXI-S	28.47 ± 7.16
STAXI-T	28.87 ± 6.88
Anger Control	19.83 ± 4.24
Anger-Out	19.00 ± 5.12
Anger-In	22.90 ± 4.74
AAQ-II	39.70 ± 9.58
EQ-5D-5L Index	0.75 (0.59–0.82)
EQ-5D-5L VAS	55.17 ± 18.45

**Abbreviations**: AAQ-II, Acceptance and Action Questionnaire-II; BDI-II, Beck Depression Inventory-II; BMI, body mass index; EQ-5D-5L, EuroQol 5-dimension 5-level; HB, Hwa-byung; HBPS, Hwa-byung Personality Scale; HBSS, Hwa-byung Symptom Scale; IQR, interquartile range; SD, standard deviation; STAI-S/-T, State-Trait Anxiety Inventory state/trait subscale; STAXI-S/-T, State-Trait Anger Expression Inventory state/trait subscale. **Note**. Values are presented as *n* (%), mean ± SD, or median (IQR) based on the Shapiro–Wilk test. †, data presented as median (interquartile range) based on the Shapiro–Wilk test.

**Table 2:** UX survey results at Week 4.

Item	Description	*N*	Mean ± SD	Median (IQR)	Positive Rate (%)
1	Week 1 video content: understandable and helpful	29	3.00 ± 0.93	3.00 (3.00–4.00)	75.9
2	Week 2 video content: understandable and helpful	29	3.14 ± 0.69	3.00 (3.00–4.00)	82.8
3	Week 3 video content: understandable and helpful	29	3.14 ± 0.69	3.00 (3.00–4.00)	82.8
4	Week 4 video content: understandable and helpful	29	3.24 ± 0.64	3.00 (3.00–4.00)	89.7
5	Diaphragmatic breathing guidance: effective	29	3.07 ± 1.00	3.00 (2.00–4.00)	72.4
6	Diaphragmatic breathing guidance: ease of use	29	2.93 ± 1.07	3.00 (2.00–4.00)	72.4
7	Relaxation therapy: effective	29	3.03 ± 0.91	3.00 (3.00–4.00)	79.3
8	Relaxation therapy: ease of use	29	2.97 ± 1.02	3.00 (2.00–4.00)	72.4
9	Meditation therapy: effective	29	3.07 ± 0.84	3.00 (3.00–4.00)	86.2
10	Meditation therapy: ease of use	29	3.07 ± 0.92	3.00 (3.00–4.00)	79.3
11	Diaphragmatic breathing (phone on abdomen): ease of use	29	2.66 ± 1.04	2.00 (2.00–4.00)	48.3
12	Diaphragmatic breathing: breath count accuracy	29	2.21 ± 1.15	2.00 (1.00–3.00)	41.4
13	Diaphragmatic breathing: ease of following instructions	29	2.72 ± 1.16	3.00 (2.00–4.00)	62.1
14	Three-line diary: ease of writing	29	3.10 ± 0.94	3.00 (3.00–4.00)	75.9
15	Three-line diary: helpful for mental health	29	3.14 ± 0.83	3.00 (3.00–4.00)	79.3
16	Three-line diary: barriers to consistent use [reverse]	29	1.79 ± 1.29	2.00 (1.00–3.00)	-
17	HB self-assessment: helpful for monitoring	29	3.14 ± 0.64	3.00 (3.00–4.00)	86.2
20	In-app guidance: clear and easy to understand	28	3.29 ± 0.71	3.00 (3.00–4.00)	85.7
21	Following usage instructions: ease	28	2.89 ± 0.99	3.00 (2.00–4.00)	60.7
22	Login process: ease	28	3.32 ± 1.02	4.00 (2.75–4.00)	75.0
Internal Consistency	Overall UX questionnaire (19 items)	27	-	-	α = 0.9067

**Abbreviations**: HB, Hwa-byung; IQR, interquartile range; SD, standard deviation; UX, user experience. **Note**. Items rated on a 0–4 Likert scale (0 = not at all, 4 = strongly agree) unless otherwise noted. Items 18 and 19 assessed usage frequency and reasons for non-daily use as categorical and multiple-response items, respectively; these data are presented separately in Table S5. Positive response rate defined as score ≥ 3 (score ≤ 1 for item 16 [reverse-scored]). Domain scores are means of constituent items. Overall and domain-level internal consistency (Cronbach’s α) are reported in the text and Table S5, respectively.

**Table 3:** Clinical scale changes from baseline to Week 4 in the mITT population.

Category	Variable	*N*	Mean ± SD (Week 0)	Mean ± SD (Week 4)	Change (%)	*p* (FDR)	Effect Size (Label)
HB Scales	HB Personality	28	47.857 ± 9.951	38.714 ± 9.257	−19.10	0.001 *	−0.791 (Cohen’s d)
	HB Symptom	28	47.107 ± 8.075	37.286 ± 8.675	−20.85	<0.001 *	−0.920 (Cohen’s d)
Psychology	BDI-II	28	38.893 ± 10.546	25.607 ± 10.577	−34.16	<0.001 *	−1.108 (Cohen’s d)
	STAI-S	28	45.536 ± 4.418	43.929 ± 4.929	−3.53	0.155	−0.311 (Cohen’s d)
	STAI-T	28	48.107 ± 5.473	43.286 ± 4.487	−10.02	<0.001 *	−0.868 (Cohen’s d)
	STAXI-S	28	28.893 ± 7.120	21.071 ± 7.537	−27.07	<0.001 *	−0.958 (Cohen’s d)
	STAXI-T	28	29.429 ± 6.691	22.857 ± 6.496	−22.33	0.001 *	−0.832 (Cohen’s d)
	Anger Control	28	19.786 ± 4.375	18.500 ± 3.776	−6.50	0.307	0.215 (r)
	Anger-Out	28	19.357 ± 4.931	16.214 ± 4.810	−16.24	0.002 *	−0.712 (Cohen’s d)
	Anger-In	28	22.929 ± 4.906	19.036 ± 4.534	−16.98	0.002 *	−0.698 (Cohen’s d)
	AAQ-II	28	40.393 ± 9.512	33.357 ± 7.445	−17.42	0.001 *	−0.779 (Cohen’s d)
QoL	EQ-5D-5L Index	28	0.682 ± 0.166	0.766 ± 0.106	+12.24	0.007 *	0.538 (r)
	EQ-5D-5L VAS	28	53.571 ± 18.046	67.143 ± 13.569	+25.33	0.002 *	0.705 (Cohen’s d)
HRV	SDNN (ms)	27	30.556 ± 13.899	28.037 ± 13.239	−8.24	0.686	0.088 (r)
	RMSSD (ms)	27	24.889 ± 16.867	21.778 ± 11.862	−12.50	0.592	0.122 (r)
	PSI	27	5.951 ± 0.661	6.096 ± 0.647	+2.43	0.307	0.224 (Cohen’s d)
	ln(TP) [ms2]	27	6.919 ± 0.514	6.768 ± 0.377	−2.18	0.155	0.310 (r)
	LF/HF	27	2.022 ± 2.773	1.989 ± 1.772	−1.65	0.761	0.059 (r)

**Abbreviations**: AAQ-II, Acceptance and Action Questionnaire-II; BDI-II, Beck Depression Inventory-II; EQ-5D-5L, EuroQol 5-dimension 5-level; FDR, false discovery rate; HB, Hwa-byung; HBPS, Hwa-byung Personality Scale; HBSS, Hwa-byung Symptom Scale; HRV, heart rate variability; LF/HF, low-frequency/high-frequency ratio; mITT, modified intention-to-treat; PSI, physical stress index; RMSSDs, root mean square of successive differences; SD, standard deviation; SDNNs, standard deviation of normal-to-normal intervals; STAI, State-Trait Anxiety Inventory; STAXI, State-Trait Anger Expression Inventory; TP, total power. **Note**. Paired *t*-test used for normally distributed differences; Wilcoxon signed-rank test for non-normal distributions. Effect size: Cohen’s d (paired *t*-test) or r (Wilcoxon). P (FDR): Benjamini–Hochberg false discovery rate correction applied across all 18 scales. *, FDR-adjusted *p* < 0.05.

## Data Availability

The data that support the findings of this study are available from the corresponding author upon reasonable request.

## Supplementary Materials

The following supporting information can be downloaded at: https://www.mdpi.com/article/10.3390/healthcare14132027/s1. Figure S1: UX survey response distribution at Week 4 (*n* = 29); Figure S2: HRV changes from baseline to Week 4 (mITT, *n* = 28); Figure S3: Exploratory Association Analysis: Concurrent Changes in Psychological Flexibility (ΔAAQ-II) and HB Symptoms (ΔHBSS); Table S1. Concurrent medications and treatments reported by enrolled participants (*n* = 30); Table S2: Missing data summary by assessment timepoint and key variable; Table S3: Feasibility and adherence metrics over the 28-day intervention period; Table S4: Adverse events (AEs) by assessment period and causality assessment; Table S5: UX domain scores, internal consistency, and usage patterns; Table S6: Repeated measures analysis: Friedman test and Dunn’s post hoc comparisons; Table S7: Changes in HB-VAS 15-item scores from baseline to Week 4; Table S8: Sensitivity analyses for clinical scale changes from baseline to Week 4; Table S9: Clinical scale and HB-VAS changes at follow-up (Week 8); Table S10: HB screening status (HBSS ≥ 30) across assessment timepoints (mITTs); Table S11: Mixed model for repeated measures (MMRMs) results for six primary clinical scales (mITT); Table S12: Hwa-byung Symptom Scale exploratory responder analysis (mITT); Table S13: Exploratory mediation path estimates and bootstrap indirect effect; Table S14: Adherence–outcome correlation: Spearman’s ρ between app use days (minimum and maximum) and change in clinical outcomes from baseline to Week 4 (mITT).

## Abbreviations

The following abbreviations are used in this manuscript:

AAQ-II	Acceptance and Action Questionnaire-II
ACT	Acceptance and Commitment Therapy
AE	Adverse Event
BDI-II	Beck Depression Inventory-II
BMI	Body Mass Index
CI	Confidence Interval
CONSORT	Consolidated Standards of Reporting Trials
CRIS	Clinical Research Information Service
DTx	Digital Therapeutics
EQ-5D-5L	EuroQol 5-Dimension 5-Level
FDR	False Discovery Rate
HB	Hwa-Byung
HBDIS	Hwa-Byung Diagnostic Interview Schedule
HBPS	Hwa-Byung Personality Scale
HBSS	Hwa-Byung Symptom Scale
HRV	Heart Rate Variability
IQR	Interquartile Range
KM	Korean Medicine
LF/HF	Low-Frequency/High-Frequency ratio
MCID	Minimally Clinically Important Difference
mITT	Modified Intention-To-Treat
MMRM	Mixed Models for Repeated Measures
PP	Per-Protocol
PSI	Physical Stress Index
RCT	Randomized Controlled Trial
RMSSD	Root Mean Square of Successive Differences
SD	Standard Deviation
SDNN	Standard Deviation of Normal-to-Normal intervals
STAI	State-Trait Anxiety Inventory
STAXI	State-Trait Anger Expression Inventory
TP	Total Power
UX	User Experience
VAS	Visual Analogue Scale
