# Supplementary material for: Digital Therapeutic for Hwa-Byung (Korean Culture-Related Anger Syndrome) Based on Acceptance and Commitment Therapy: A Pilot Feasibility Trial
Source: Healthcare (Basel). 2026 Jul 7;14(13):2027. doi: 10.3390/healthcare14132027 (PMC13362309; doi:10.3390/healthcare14132027)
Supplement: Supplementary file 1 [file healthcare-14-02027-s001.zip › healthcare-4333770-supplementary.pdf]

Figure S1. UX survey response distribution at Week 4 (n = 29).

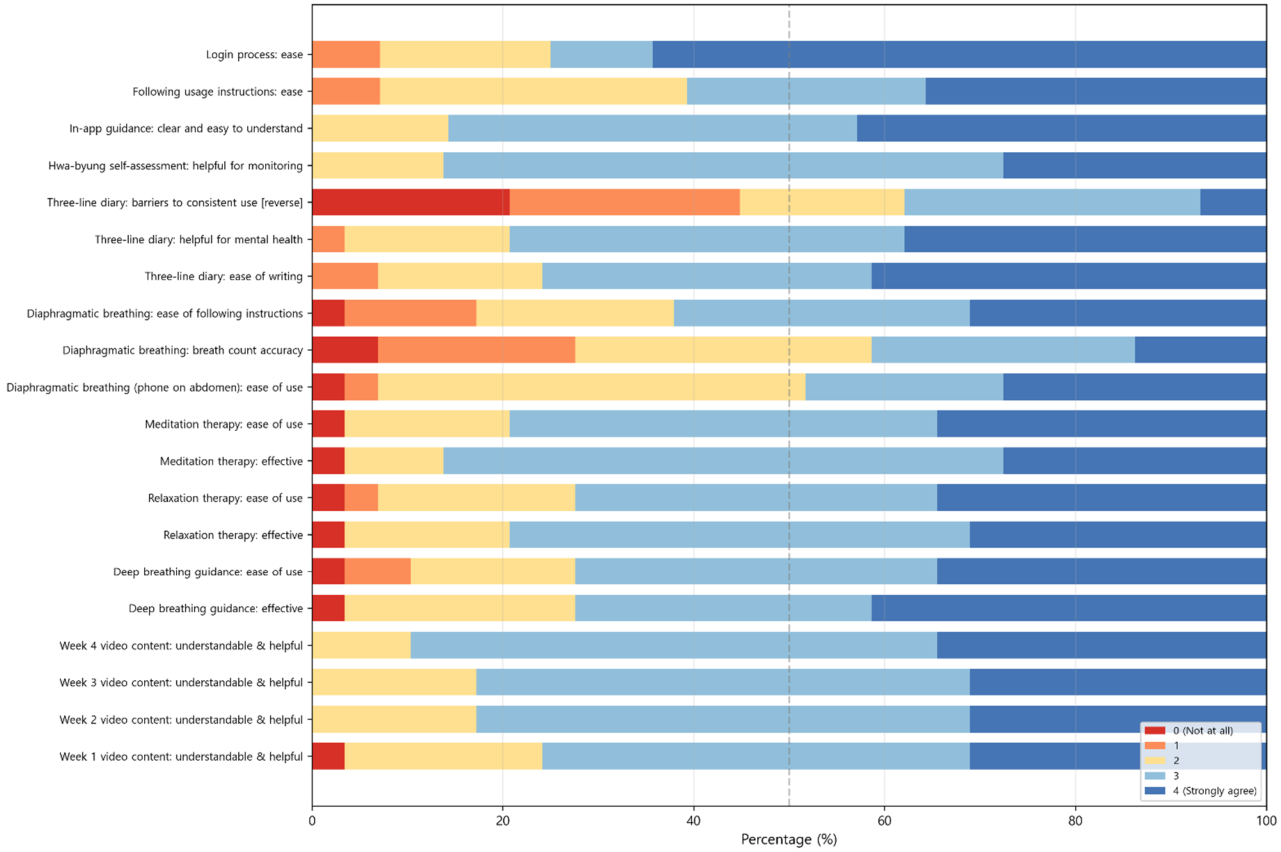

Note. Stacked horizontal bar chart showing percentage of respondents selecting each Likert score (0–4) per item. Items ordered as in the questionnaire. Item 16 (reverse-scored) indicated.

Figure S2. HRV changes from baseline to Week 4 (mITT, n=28).

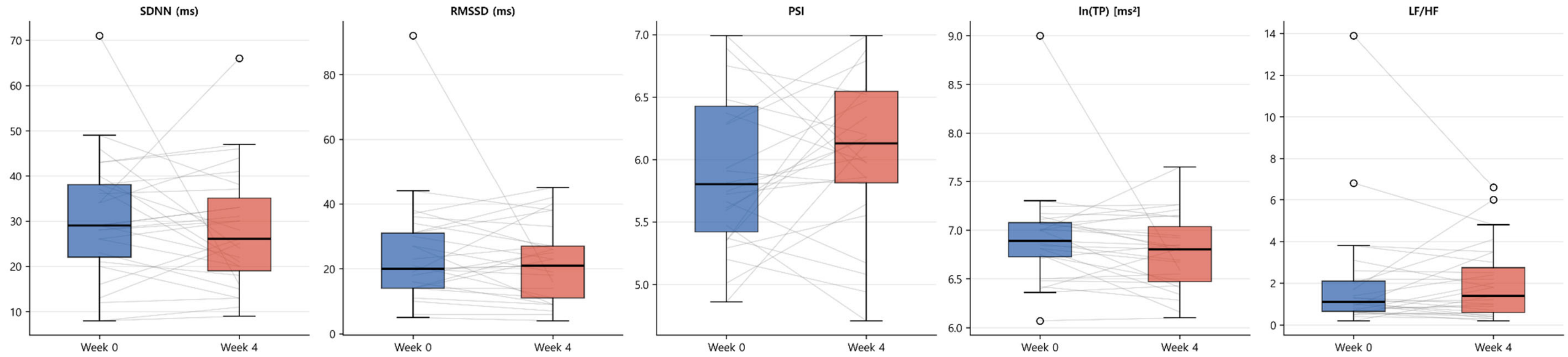

Note. Box plots with paired individual trajectories. None of the HRV parameters showed statistically significant change (all FDR-adjusted p > 0.05).

Figure S3. Exploratory Association Analysis: Concurrent Changes in Psychological Flexibility (ΔAAQ-II) and HB Symptoms (ΔHBSS).

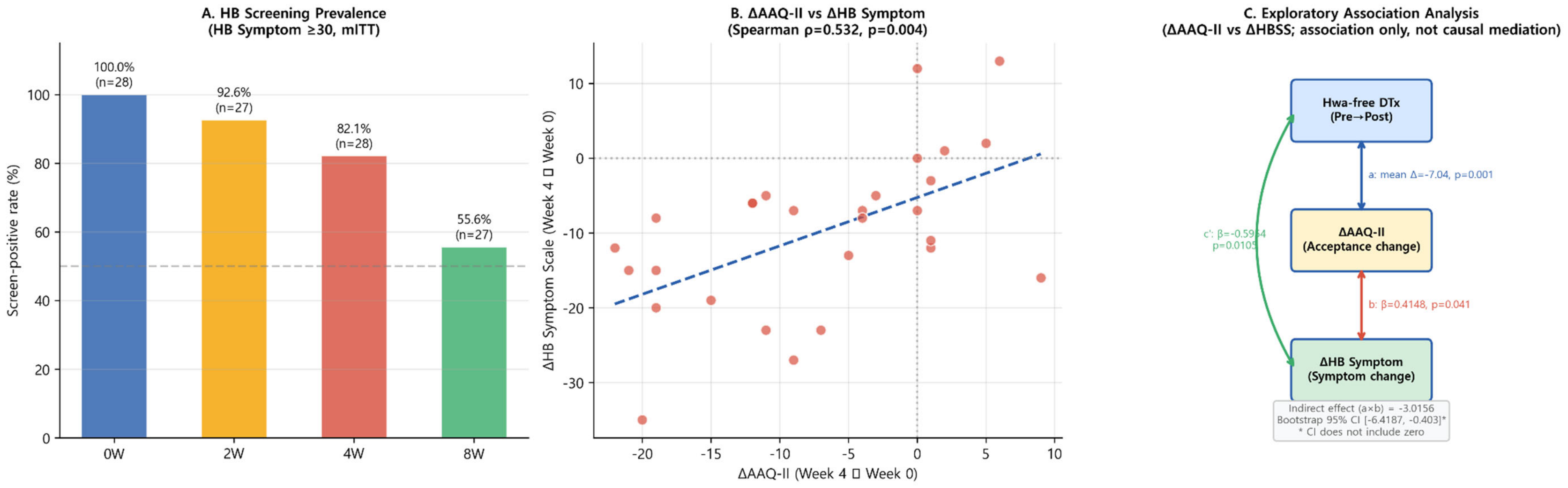

Note. Panel A: HB screening-positive rate across timepoints (mITT). Panel B: Scatter plot of ΔAAQ-II versus ΔHBSS with Spearman correlation. Panel C: Mediation path diagram (Baron & Kenny framework with bootstrap). All findings are exploratory; causal inference is not warranted from a single-arm design. ΔAAQ-II = change in Acceptance and Action Questionnaire-II (negative = improvement); ΔHBSS = change in Hwa-byung Symptom Scale (negative = improvement).

Table S1. Concurrent medications and treatments reported by enrolled participants (n = 30).

ID	Concurrent medication	Details
1	No	None
2	No	None
3	Yes	Osteoporosis (Admin Plus Tab, PO); Dyslipidaemia (Crestor Tab 10 mg, PO)
4	Yes	Hypertension (Norvasc Tab 5 mg, PO); Hypertension (Olmesartan 40 mg, PO); Dyslipidaemia (Lipitor Tab 10 mg, PO)
5	Yes	Dyslipidaemia (Lipitor Tab 10 mg, PO); Osteoporosis (Cavid Chewable, PO); Obesity (Mounjaro, SC)
6	Yes	Indigestion (Korean herbal formula Cheongwidan, PO); Menopausal symptoms (Livial 2.5 mg, PO); Hypothyroidism (Synthyroid Tab 0.025 mg, PO); Migraine (Craming Tab, PO); Migraine (Tylenol ER 650 mg, PO); Migraine (Indenol Tab 40 mg, PO)
7	Yes	Diabetes mellitus (Jardiance Duo Tab 12.5/500 mg, PO)
8	No	None
9	No	None
10	No	None
11	No	None
12	Yes	Diabetes mellitus (Dynav ER Tab, PO); Diabetes mellitus (Tenelia Tab, PO)
13	No	None
14	Yes	Hypertension (Novdipine S Tab 2.5 mg, PO); Chronic tonsillitis (Muteran Cap 200 mg, PO); Chronic tonsillitis (Trilone Tab, PO); Chronic tonsillitis (Pelubi S Tab, PO); Chronic tonsillitis (Jaqbo Tab 20 mg, PO); Chronic tonsillitis (Stogar Tab, PO); Chronic tonsillitis (Dicknol Inj 90 mg/6 mL, IM); Chronic tonsillitis (Cough Syr 20 ml/pk, PO)
15	No	None
16	No	None
17	Yes	Hypertension (Twinheart Tab 40/5 mg, PO); Dyslipidaemia (Omaron Soft Cap, PO); Arteriosclerosis (Clopina Tab, PO); Angina pectoris (Metazen-MR Tab 35 mg, PO)

18	Yes	Hypertension (Dichlozid Tab 25 mg, PO); Reflux oesophagitis (Arlovo Tab 25 mg, PO); Reflux oesophagitis (Repadrin Tab 100 mg, PO)
19	Yes	Dyslipidaemia (Rosuzet Tab 10/2.5 mg, PO)
20	No	None
21	Yes	Acute nasopharyngitis (Rino-Ebastel 10/120 mg, PO); Acute nasopharyngitis (Actifed tablet 60 mg, PO); Acute nasopharyngitis (Xyzal 5 mg, PO); Acute nasopharyngitis (Antax Cap 180 mg, PO); Acute nasopharyngitis (Tyrenol ER 650 mg, PO); Allergic dry eye syndrome (Hyalon Eye Drops 0.15% 0.45 mL, EXT)
22	Yes	Generalised myalgia (Korean herbal formula Cheontong-hwan, PO)
23	No	None
24	No	None
25	No	None
26	Yes	Insomnia (Zolmin Tab 0.25 mg [triazolam], PO)
27	No	None
28	Yes	Hypertension (Closartan 50/125 mg, PO)
29	Yes	Dyslipidaemia (Lipitor Tab 10 mg, PO)
30	No	None

Note. Concurrent medications: pre-existing treatments stable for ≥4 weeks prior to enrolment.

Table S2. Missing data summary by assessment timepoint and key variable.

Timepoint	Variable	N of missing	%	N of available
**0W**	AAQ-II	0	0	30
	Anger-Control	0	0	30
	Anger-In	0	0	30
	Anger-Out	0	0	30
	BDI-II	0	0	30
	EQ-5D-5L	0	0	30
	HB Personality	0	0	30
	HB Symptom	0	0	30
	HB-VAS 15 items	0	0	30
	HRV	1	3.3	29
	STAI-S	0	0	30
	STAI-T	0	0	30
	STAXI-S	0	0	30
	STAXI-T	0	0	30
**2W**	AE	2	6.7	28
	Anger-Control	2	6.7	28
	Anger-In	2	6.7	28
	Anger-Out	2	6.7	28
	BDI-II	2	6.7	28
	HB Personality	2	6.7	28
	HB Symptom	2	6.7	28
	HB-VAS 15 items	2	6.7	28
	STAI-S	2	6.7	28
	STAI-T	2	6.7	28
	STAXI-S	2	6.7	28
	STAXI-T	2	6.7	28
**4W**	AAQ-II	1	3.3	29
	AE	1	3.3	29
	Anger-Control	1	3.3	29
	Anger-In	1	3.3	29
	Anger-Out	1	3.3	29
	BDI-II	1	3.3	29
	EQ-5D-5L	1	3.3	29
	HB Personality	1	3.3	29
	HB Symptom	1	3.3	29
	HB-VAS 15 items	1	3.3	29
	HRV_PSI	2	6.7	28

	STAI-S	1	3.3	29
	STAI-T	1	3.3	29
	STAXI-S	1	3.3	29
	STAXI-T	1	3.3	29
	UX questionnaire	1	3.3	29
	Use data (min. use, max. use)	1	3.3	29
**8W**	AAQ-II	2	6.7	28
	Anger-Control	2	6.7	28
	Anger-In	2	6.7	28
	Anger-Out	2	6.7	28
	BDI-II	2	6.7	28
	EQ-5D-5L	2	6.7	28
	HB Personality	2	6.7	28
	HB Symptom	2	6.7	28
	HB-VAS 15 items	2	6.7	28
	STAI-S	2	6.7	28
	STAI-T	2	6.7	28
	STAXI-S	2	6.7	28
	STAXI-T	2	6.7	28

Table S3. Feasibility and adherence metrics over the 28-day intervention period.

Metric	N	Mean ± SD	Median (IQR)	Min	Max	Adherence rate (Mean ± SD)
Minimum use days (app launch)	28	20.32 ± 7.78	22.50 (12.75–27.00)	4.00	28.00	72.58 ± 27.79%
Maximum use days (full session)	28	11.89 ± 9.47	15.00 (2.50–18.50)	0.00	27.00	42.47 ± 33.82%
Incomplete session days (Min − Max)	28	8.43 ± 5.88	6.50 (4.00–11.25)	1.00	22.00	-
Mean daily usage time (min)	28	26.89 ± 18.33	23.60 (13.47–32.75)	5.70	89.90	-
Cumulative total usage time (min)	28	536.31 ± 470.92	450.05 (220.00–706.25)	84.90	2516.40	-
ACT mandatory session completions (max 12)	28	10.00 ± 2.00	9.50 (9.00–12.00)	6.00	12.00	-
ACT mandatory session mean duration (min)	28	2.80 ± 0.30	2.90 (2.70–2.95)	2.10	3.20	-
ACT review session completions	28	15.04 ± 22.74	3.50 (1.00–19.25)	0.00	91.00	-
Diaphragmatic breathing module uses (occasions)	28	12.39 ± 9.70	15.00 (2.50–20.50)	0.00	27.00	-
Relaxation therapy/meditation module uses (occasions)	28	5.07 ± 9.75	1.00 (0.00–5.75)	0.00	47.00	-
Maximum consecutive use days	28	14.71 ± 9.93	12.00 (6.00–24.25)	1.00	28.00	-
Maximum consecutive non-use days	28	3.25 ± 4.09	1.50 (0.75–4.25)	0.00	13.00	-

Note. Engagement data derived from app monitoring system (mITT, n = 28). Min. use = app launch with access to ≥1 feature; Full session = completion of the mandatory diaphragmatic breathing module. ACT mandatory sessions: 3 sessions/week × 4 weeks = maximum 12 sessions; 12 of 28 participants (42.9%) completed all 12 mandatory sessions. The most common usage time window was 22:00–02:00 (n = 15, 53.6%), followed by 18:00–22:00 (n = 8, 28.6%), 10:00–14:00 (n = 3, 10.7%), and 02:00–06:00 and 06:00–10:00 (n = 1 each, 3.6%). One participant's engagement data were unavailable due to an ad-blocking DNS configuration that prevented server-side data transmission; this participant was excluded from the mITT population.

Table S4. Adverse events (AEs) by assessment period and causality assessment.

Period	Participants with ≥1 AE, n (%)	Total AE events, n	AE description	Causality
**Week 0–2**	2 (6.7)	3	Insomnia onset	Probably not related
			Chronic tonsillitis	Probably not related
			Pre-hypertension	Definitely not related
**Week 2–4**	3 (10.0)	5	Hypertension	Definitely not related
			Reflux esophagitis	Definitely not related
			Acute nasopharyngitis	Definitely not related
			Allergic dry eye syndrome	Definitely not related
			Generalised myalgia	Probably not related

Note. n (%) denotes number of participants experiencing at least one AE; total AE events may exceed participant count where one participant reported multiple AEs. AE descriptions are reported as assessed by the clinical investigator; MedDRA coding was not applied in this pilot study. No serious AEs were reported. Causality assessed per ICH E2A guidelines.

Table S5. UX domain scores, internal consistency, and usage patterns.

Part A. UX Domain Scores and Internal Consistency (N = 29)

Domain	Items (n)	Mean ± SD	Median (IQR)	Cronbach's α	Interpretation
**Video Content**	1–4 (4)	3.13 ± 0.63	3.00 (2.75–3.75)	0.8706	Good
**Diaphragmatic Breathing**	5, 6, 11–13 (5)	2.72 ± 0.95	2.60 (2.20–3.40)	0.9235	Excellent
**Relaxation Therapy**	7, 8 (2)	3.00 ± 0.94	3.00 (2.50–4.00)	0.9402	Excellent
**Meditation Therapy**	9, 10 (2)	3.07 ± 0.82	3.00 (3.00–3.50)	0.8410	Good
**Three-line Diary**	14, 15 (2)	3.12 ± 0.78	3.00 (3.00–4.00)	0.6885	Questionable
**Self-Assessment**	17 (1)	3.14 ± 0.64	3.00 (3.00–4.00)	-	-
**App Usability***	20–22 (3)	3.13 ± 0.79	3.33 (2.33–4.00)	0.7835	Acceptable

Note. *, n = 27 due to missing data on item 22.

Part B. Usage Frequency and Reasons for Non-daily Use

Usage Frequency (Item 18)	n (%)	Reasons for non-daily use (Item 19)†	n (% of respondents)
Daily	13 (44.8)	Insufficient reminders/alerts	9 (42.9)
4–5 times/week	9 (31.0)	Content too long or burdensome	5 (23.8)
2–3 times/week	5 (17.2)	Did not feel immediate need	2 (9.5)
Rarely	2 (6.9)	Difficult to access features	2 (9.5)
		Other	6 (28.6)

Note. †, Multiple responses allowed (N = 21 respondents who did not use the app daily).

Table S6. Repeated measures analysis: Friedman test and Dunn's post-hoc comparisons.

Part A. mITT Analysis (n = 26–27)

Category	Variable	χ2	p-value	0W (Mean±SD)	2W (Mean±SD)	4W (Mean±SD)	8W (Mean±SD)	Post-hoc Sig. (Dunn)
HB Scales	HB Personality	41.44	<.001	48.58±9.68	40.81±9.72	37.54±8.43	35.23±7.19	0W>4W, 8W
	HB Symptom	35.44	<.001	47.73±8.03	41.19±8.58	36.77±8.63	31.08±10.51	0W>4W, 8W; 2W>8W
Psychology	BDI-II	36.13	<.001	39.92±10.18	30.58±8.56	25.23±10.49	22.31±9.95	0W>4W, 8W; 2W>8W
	STAI-S	17.09	<.001	45.42±4.57	42.65±5.00	43.27±4.41	40.81±2.70	0W>8W
	STAI-T	26.18	<.001	48.23±5.62	42.65±4.60	42.92±4.42	41.50±5.09	0W>2W, 4W, 8W
	STAXI-S	35.76	<.001	29.50±6.94	24.04±7.90	21.12±7.25	17.00±7.18	0W>4W, 8W; 2W>8W
	STAXI-T	30.23	<.001	29.92±6.39	24.35±5.75	22.58±6.32	20.50±6.53	0W>2W, 4W, 8W
	Anger Control	5.76	.124	19.42±4.27	18.38±3.44	18.38±3.81	17.62±3.15	None
	Anger-Out	20.80	<.001	19.62±5.02	17.46±4.96	16.35±4.81	15.15±4.23	0W>8W
	Anger-In	27.20	<.001	23.15±4.99	19.88±4.22	18.85±3.59	17.19±3.72	0W>4W, 8W
	AAQ-II	27.08	<.001	41.19±8.70	-	33.85±7.10	27.81±7.30	0W>4W, 8W; 4W>8W
QoL	EQ-5D Index	16.64	<.001	0.68±0.17	-	0.77±0.11	0.82±0.09	0W<8W
	EQ-5D VAS	12.02	.003	54.07±18.19	-	66.30±13.05	72.37±10.79	0W<8W

Note. Within the modified intention-to-treat (mITT) population (n=28), the sample size for the Friedman test ranged from 26 to 27 due to the requirement for complete data across all analyzed time points. For variables analyzed at three time points (0W, 4W, and 8W), such as AAQ-II and EQ-5D, one participant was excluded due to missing data at the 8W follow-up, resulting in n=27. For other clinical scales requiring assessments at four time points (0W, 2W, 4W, and 8W), two participants were excluded due to missing data at either the 2W or 8W visits, resulting in n=26. This discrepancy reflects the overlap of missing assessments at 2W and 8W among the mITT participants.

Part B. mITT Analysis: HB-VAS 15 Items (n = 26)

No	Symptom	χ2	p-value	0W (Mean±SD)	2W (Mean±SD)	4W (Mean±SD)	8W (Mean±SD)	Post-hoc Sig. (Dunn)
**01**	**Chest tightness/breathlessness**	22.58	<.001	74.08 ± 19.74	68.35 ± 19.45	60.77 ± 22.08	57.00 ± 22.01	0W>8W
**02**	**Rising sensation**	28.26	<.001	76.65 ± 18.14	72.00 ± 17.43	63.12 ± 18.85	56.58 ± 23.21	0W>4W, 8W
**03**	**Heat sensation (face/chest)**	27.73	<.001	75.19 ± 19.81	70.27 ± 19.72	60.42 ± 23.86	50.69 ± 25.92	0W>8W; 2W>8W
**04**	**Lump sensation (throat/epigastrium)**	29.56	<.001	77.96 ± 21.22	68.15 ± 23.72	60.23 ± 20.62	52.31 ± 24.96	0W>4W, 8W
**05**	**Feeling of injustice/resentment**	24.41	<.001	78.27 ± 23.54	70.85 ± 22.36	61.54 ± 21.85	54.58 ± 25.20	0W>4W, 8W
**06**	**Accumulated anger/rage**	30.43	<.001	83.77 ± 18.43	74.88 ± 18.71	66.73 ± 21.54	57.27 ± 24.21	0W>4W, 8W
**07**	**Palpitations**	23.23	<.001	74.12 ± 22.24	67.12 ± 16.56	60.50 ± 20.60	49.35 ± 26.78	0W>8W
**08**	**Sleep disturbance**	17.50	.001	84.77 ± 18.33	77.12 ± 19.03	68.12 ± 20.40	67.46 ± 21.76	0W>4W, 8W
**09**	**Headache/dizziness**	34.13	<.001	76.38 ± 22.66	65.73 ± 20.31	56.81 ± 21.31	47.54 ± 25.51	0W>4W, 8W
**10**	**Dry mouth**	23.05	<.001	73.00 ± 25.19	64.19 ± 20.85	58.83 ± 24.38	49.12 ± 26.01	0W>8W
**11**	**Loss of appetite**	19.63	<.001	60.38 ± 26.04	50.38 ± 23.75	45.87 ± 23.25	37.58 ± 26.25	0W>8W
**12**	**Fearfulness/startle response**	27.25	<.001	75.00 ± 23.52	69.42 ± 21.58	58.73 ± 22.85	50.54 ± 29.38	0W>8W
**13**	**Intrusive thoughts**	25.76	<.001	85.58 ± 16.19	80.77 ± 16.04	72.42 ± 17.32	61.58 ± 26.84	0W>4W, 8W; 2W>8W
**14**	**Frequent sighing**	29.92	<.001	84.00 ± 15.29	75.08 ± 21.20	68.85 ± 20.88	54.58 ± 26.92	0W>4W, 8W; 2W>8W
**15**	**Deep-seated resentment (Han)**	23.82	<.001	81.85 ± 20.77	75.27 ± 19.21	63.38 ± 23.66	54.88 ± 27.19	0W>4W, 8W; 2W>8W

Note. Within the modified intention-to-treat (mITT) population (n=28), the sample size for the Friedman test was n=26 for the HB-VAS 15 items requiring assessments at four time points (0W, 2W, 4W, and 8W). Two participants were excluded because they failed to provide complete data at either the 2W or 8W visits, reflecting the overlap of missing assessments within the mITT group. Post-hoc comparisons were performed using Dunn's test with Bonferroni correction.

Part C. PP Analysis (Sensitivity, n = 17–18)

Category	Variable	χ2	p-value	0W (Mean±SD)	2W (Mean±SD)	4W (Mean±SD)	8W (Mean±SD)	Post-hoc Sig. (Dunn)
**HB Scales**	HB Personality	24.94	<.001	47.71±8.36	39.18±8.97	37.53±8.52	34.47±7.88	0W>4W, 8W
	HB Symptom	22.15	<.001	47.06±7.55	40.41±8.56	35.71±9.66	28.71±11.22	0W>4W, 8W; 2W>8W
**Psychology**	BDI-II	20.36	<.001	39.35±10.64	30.00±9.53	23.76±12.43	21.00±10.56	0W>4W, 8W
	STAI-S	8.47	.037	45.53±5.35	42.00±4.49	42.12±3.89	40.71±3.04	0W>8W
	STAI-T	16.22	.001	48.00±5.99	42.29±4.50	42.29±4.67	41.35±5.33	0W>8W
	STAXI-S	16.37	.001	28.53±7.73	22.94±8.75	20.94±8.07	17.47±7.71	0W>8W
	STAXI-T	16.35	.001	28.71±6.92	23.00±5.83	21.35±7.13	20.29±7.03	0W>4W, 8W
	Anger Control	3.84	.279	19.29±4.55	18.82±3.28	18.12±4.26	18.24±3.42	None
	Anger-Out	8.75	.033	18.41±5.03	16.41±4.51	15.71±5.22	14.82±4.57	None
	Anger-In	11.07	.011	22.35±5.31	19.35±3.95	18.94±4.19	17.47±4.33	0W>8W
	AAQ-II	17.94	<.001	40.17±9.75	-	33.00±7.79	26.61±7.21	0W>8W
**QoL**	EQ-5D Index	12.45	.002	0.68±0.18	-	0.77±0.10	0.83±0.11	0W<8W
	EQ-5D VAS	10.39	.006	51.94±16.28	-	62.50±12.28	72.78±10.32	0W<8W

Note. In the per-protocol (PP) population (n=19), the sample size for the Friedman test varied between n=17 and n=18 due to the requirement for complete data across all analyzed time points. For variables measured at three time points (0W, 4W, and 8W), such as AAQ-II and EQ-5D, one participant was excluded due to a missing 8W follow-up visit, resulting in n=18. For clinical scales measured at four time points (0W, 2W, 4W, and 8W), an additional participant who missed the 2W assessment was excluded along with a participant, resulting in n=17. This individual was one of the two participants with missing 2W data reported in the attrition analysis (**Table S1**) who otherwise met the PP criteria.

Part D. PP Analysis: HB-VAS 15 Items (Sensitivity, n = 17)

No	Symptom	χ2	p-value	0W (Mean±SD)	2W (Mean±SD)	4W (Mean±SD)	8W (Mean±SD)	Post-hoc Sig. (Dunn)

**01**	**Chest tightness/breathlessness**	12.23	.007	70.88±18.56	63.82±16.91	56.00±20.90	51.76±22.84	0W > 8W
**02**	**Rising sensation**	17.67	<.001	73.65±18.89	69.12±17.16	60.76±19.09	50.88±24.64	0W > 8W
**03**	**Heat sensation (face/chest)**	15.81	.001	71.71±21.20	67.94±19.69	59.00±22.53	47.53±24.62	0W > 8W
**04**	**Lump sensation (throat/epigastrium)**	18.98	<.001	77.12±20.87	66.18±21.76	59.29±17.82	47.71±24.11	0W > 8W
**05**	**Feeling of injustice/resentment**	17.34	.001	75.18±26.10	69.12±22.10	59.82±24.15	49.71±25.03	0W > 8W
**06**	**Accumulated anger/rage**	18.00	<.001	82.41±19.56	72.65±18.72	63.35±22.76	53.53±25.17	0W > 8W
**07**	**Palpitations**	18.51	<.001	69.53±24.15	63.53±16.37	53.53±19.59	44.06±27.57	0W > 8W
**08**	**Sleep disturbance**	12.40	.006	79.59±20.18	70.29±19.40	60.18±18.99	60.47±21.05	0W > 4W
**09**	**Headache/dizziness**	27.86	<.001	76.88±21.44	62.35±20.16	53.82±20.58	44.12±21.88	0W > 4W, 8W
**10**	**Dry mouth**	13.40	.004	74.35±23.79	62.65±19.21	56.44±23.09	43.35±22.79	0W > 8W
**11**	**Loss of appetite**	11.61	.009	59.71±24.01	50.29±22.18	45.44±20.92	35.29±26.31	0W > 8W
**12**	**Fearfulness/startle response**	17.91	<.001	72.12±23.51	65.94±19.77	54.53±20.83	39.65±26.11	0W > 8W; 2W > 8W
**13**	**Intrusive thoughts**	17.28	.001	83.82±15.75	78.35±15.47	69.47±16.29	58.82±22.81	0W > 8W; 2W > 8W
**14**	**Frequent sighing**	14.98	.002	82.47±14.89	71.59±23.04	64.53±22.60	51.47±24.99	0W > 8W
**15**	**Deep-seated resentment (Han)**	15.91	.001	80.65±22.69	73.94±19.76	59.47±25.21	50.53±27.21	0W > 8W

Note. In the per-protocol (PP) population (n=19), the sample size for the Friedman test was n=17 for the HB-VAS 15 items requiring assessments across four time points (0W, 2W, 4W, and 8W). Exclusions included one participant due to a missing 8W follow-up visit and one additional participant due to a missing 2W assessment, resulting in a total of 17 participants with complete longitudinal data. This specific exclusion of the 2W-missing individual corresponds to the attrition details reported in **Table S1**. Post-hoc comparisons were conducted using Dunn's test with Bonferroni correction.

Table S7. Changes in HB-VAS 15-item scores from baseline to Week 4.

Part A. mITT Analysis (Primary, n = 28)

No	Symptom	Baseline	Week 4	Change	p-value (FDR)	Cohen's d
**01**	**Chest tightness/breathlessness**	73.07 ± 19.54	61.96 ± 21.71	-11.11 ± 15.04	0.0045	-0.74
**02**	**Rising sensation**	76.18 ± 18.37	64.50 ± 18.95	-11.68 ± 17.29	0.0045	-0.68
**03**	**Heat sensation (face/chest)**	75.18 ± 20.24	62.54 ± 24.38	-12.64 ± 23.68	0.0150	-0.53
**04**	**Lump sensation (throat/epigastrium)**	77.39 ± 21.23	62.71 ± 21.88	-14.68 ± 21.49	0.0045	-0.68
**05**	**Feeling of injustice/resentment**	76.25 ± 23.83	63.93 ± 22.82	-12.32 ± 27.40	0.0264	-0.45
**06**	**Accumulated anger/rage**	82.96 ± 17.99	67.86 ± 21.23	-15.11 ± 17.00	0.0015	-0.89
**07**	**Palpitations**	74.36 ± 21.43	61.18 ± 20.71	-13.18 ± 23.87	0.0150	-0.55
**08**	**Sleep disturbance**	84.07 ± 19.08	70.04 ± 20.90	-14.04 ± 23.58	0.0100	-0.60
**09**	**Headache/dizziness**	73.79 ± 23.96	59.00 ± 22.29	-14.79 ± 29.10	0.0156	-0.51
**10**	**Dry mouth**	71.36 ± 24.98	60.52 ± 24.35	-10.84 ± 29.80	0.0649	-0.36
**11**	**Loss of appetite**	60.00 ± 25.13	46.88 ± 22.84	-13.13 ± 19.61	0.0045	-0.67
**12**	**Fearfulness/startle response**	73.21 ± 23.57	60.79 ± 23.50	-12.43 ± 25.38	0.0175	-0.49
**13**	**Intrusive thoughts**	84.82 ± 17.22	73.68 ± 17.51	-11.14 ± 20.94	0.0150	-0.53
**14**	**Frequent sighing**	82.46 ± 16.12	70.71 ± 21.28	-11.75 ± 22.86	0.0156	-0.51
**15**	**Deep-seated resentment (Han)**	79.21 ± 22.25	64.75 ± 23.40	-14.46 ± 28.58	0.0156	-0.51

Note. Values are presented as mean ± SD. p-values were calculated using paired t-tests.

Part B. PP Analysis (Sensitivity, n = 19)

No	Symptom	Baseline	Week 4	Change	p-value (FDR)	Cohen's d
**01**	**Chest tightness/breathlessness**	69.74 ± 18.14	58.26 ± 20.86	-11.47 ± 17.23	0.0647	-0.67
**02**	**Rising sensation**	73.26 ± 19.05	63.05 ± 19.42	-10.21 ± 19.00	0.0660	-0.54
**03**	**Heat sensation (face/chest)**	72.05 ± 21.68	62.26 ± 23.62	-9.79 ± 24.43	0.1047	-0.40
**04**	**Lump sensation (throat/epigastrium)**	76.37 ± 20.90	63.05 ± 20.29	-13.32 ± 23.51	0.0647	-0.57
**05**	**Feeling of injustice/resentment**	72.53 ± 25.86	63.53 ± 25.38	-9.00 ± 30.75	0.2183	-0.29

**06**	**Accumulated anger/rage**	81.37 ± 18.73	65.37 ± 22.43	-16.00 ± 19.34	0.0300	-0.83
**07**	**Palpitations**	70.37 ± 22.92	55.26 ± 20.31	-15.11 ± 25.03	0.0647	-0.60
**08**	**Sleep disturbance**	79.11 ± 20.82	63.84 ± 21.07	-15.26 ± 27.41	0.0647	-0.56
**09**	**Headache/dizziness**	73.00 ± 23.56	57.37 ± 22.51	-15.63 ± 31.36	0.0723	-0.50
**10**	**Dry mouth**	71.79 ± 23.71	59.18 ± 23.40	-12.61 ± 29.76	0.1016	-0.42
**11**	**Loss of appetite**	59.21 ± 22.75	46.97 ± 20.52	-12.24 ± 21.39	0.0647	-0.57
**12**	**Fearfulness/startle response**	69.79 ± 23.23	58.00 ± 22.61	-11.79 ± 28.69	0.1040	-0.41
**13**	**Intrusive thoughts**	82.90 ± 17.25	71.63 ± 17.00	-11.26 ± 22.13	0.0723	-0.51
**14**	**Frequent sighing**	80.37 ± 15.94	67.74 ± 23.43	-12.63 ± 26.09	0.0737	-0.48
**15**	**Deep-seated resentment (Han)**	76.90 ± 24.22	61.90 ± 24.98	-15.00 ± 32.06	0.0769	-0.47

Note. Values are presented as mean ± SD. p-values were calculated using paired t-tests.

Table S8. Sensitivity analyses for clinical scale changes from baseline to Week 4.

Part A. Per-Protocol Analysis (Sensitivity, n = 19)

Category	Variable	Baseline	Week 4	Change	p-value (FDR)	Effect size
**HB Scales**	HB Personality Scale	46.74 ± 8.86	39.26 ± 9.65	-7.47 ± 12.66	0.0430	-0.59[a]
	HB Symptom Scale	46.21 ± 7.58	36.58 ± 9.68	-9.63 ± 11.64	0.0120	-0.83[a]
**Psychological Scales**	Depression (BDI-II)	37.90 ± 11.00	24.47 ± 12.42	-13.42 ± 13.55	0.0072	-0.99[a]
	State Anxiety (STAI-S)	45.68 ± 5.07	43.21 ± 4.98	-2.47 ± 5.15	0.0759	-0.48[a]
	Trait Anxiety (STAI-T)	47.84 ± 5.73	42.90 ± 4.81	-4.95 ± 5.86	0.0120	-0.84[a]
	State Anger (STAXI-S)	27.74 ± 7.78	20.90 ± 8.37	-6.84 ± 8.69	0.0135	-0.79[a]
	Trait Anger (STAXI-T)	28.11 ± 7.16	21.90 ± 7.36	-6.21 ± 9.08	0.0240	-0.68[a]
	Anger Control	19.84 ± 4.67	18.32 ± 4.18	-1.53 ± 4.33	0.1958	-0.35[a]
	Anger-Out	18.16 ± 4.81	15.58 ± 5.16	-2.58 ± 4.83	0.0596	-0.53[a]
	Anger-In	22.11 ± 5.11	19.21 ± 5.34	-2.90 ± 5.72	0.0668	-0.51[a]
	AAQ-II	39.05 ± 10.65	32.32 ± 8.14	-6.74 ± 10.22	0.0260	-0.66[a]
**Quality of Life**	EQ-5D-5L Index	0.68 ± 0.18	0.77 ± 0.10	0.09 ± 0.17	0.0596	0.49[b]
	EQ-5D-5L VAS	51.32 ± 16.06	63.95 ± 13.50	12.63 ± 16.70	0.0144	0.76[a]
**HRV**	SDNN (ms)	29.17 ± 14.34	29.06 ± 14.08	-0.11 ± 16.68	0.5819	0.16[b]
	RMSSD (ms)	25.22 ± 18.92	22.33 ± 10.72	-2.89 ± 20.15	0.9621	0.01[b]
	PSI	6.08 ± 0.65	6.04 ± 0.68	-0.03 ± 0.62	0.9195	-0.05[a]
	ln(TP) [ms^2]	6.96 ± 0.57	6.82 ± 0.35	-0.14 ± 0.62	0.5363	0.19[b]
	LF/HF	1.37 ± 1.00	1.76 ± 1.57	0.39 ± 1.41	0.9195	0.04[b]

Note. Values are presented as mean ± SD. [a], Effect size is Cohen's d for paired t-test. [b], Effect size is r for Wilcoxon signed-rank test.

Part B. Last Observation Carried Forward Analysis (mITT, n = 28)

Scale	N	N imputed	Change, 4W–0W (mean ± SD)	p (FDR-adjusted)	Effect size
**Hwa-byung Symptom Scale**	28	0	−9.82 ± 10.68	<0.001	d = −0.92
**Depression (BDI-II)**	28	0	−13.29 ± 11.99	<0.001	d = −1.11
**State Anxiety (STAI-S)**	28	0	−1.61 ± 5.17	0.112	d = −0.31
**Trait Anxiety (STAI-T)**	28	0	−4.82 ± 5.56	<0.001	d = −0.87
**State Anger (STAXI-S)**	28	0	−7.82 ± 8.17	<0.001	d = −0.96
**Trait Anger (STAXI-T)**	28	0	−6.57 ± 7.90	<0.001	d = −0.83
**AAQ-II**	28	0	−7.04 ± 9.03	<0.001	d = −0.78

Note. No Week 4 data were missing within the mITT population (n = 0 imputed for all scales); LOCF results are therefore numerically identical to the complete-case results reported in Table 3. p-values are FDR-adjusted (Benjamini–Hochberg).

..

Table S9. Clinical scale and HB-VAS changes at follow-up (Week 8).

Part A. Clinical Scale Changes (0W→8W and 4W→8W)

Variable	Analysis	0W→8W Change (Mean ± SD)	p-value (FDR)	4W→8W Change (Mean ± SD)	p-value (FDR)
HB Personality	mITT (n=27)	-13.00 ± 9.88	<0.001	-2.78 ± 7.70	0.0589
	PP (n=18)	-12.72 ± 10.20	<0.001	-3.72 ± 7.97	0.0741
HB Symptom	mITT (n=27)	-16.11 ± 12.36	<0.001	-6.04 ± 9.05	0.0108
	PP (n=18)	-17.44 ± 13.84	<0.001	-7.44 ± 8.66	0.0130
Depression (BDI-II)	mITT (n=27)	-17.07 ± 15.19	<0.001	-3.63 ± 11.56	0.1236
	PP (n=18)	-17.50 ± 17.25	0.0011	-3.83 ± 12.91	0.2921
State Anxiety	mITT (n=27)	-4.81 ± 4.73	<0.001	-3.04 ± 5.57	0.0286
	PP (n=18)	-5.11 ± 5.52	0.0018	-2.33 ± 6.37	0.2252
Trait Anxiety	mITT (n=27)	-6.70 ± 6.31	<0.001	-1.63 ± 5.15	0.1236
	PP (n=18)	-6.61 ± 6.21	0.0010	-1.28 ± 5.31	0.3485
State Anger	mITT (n=27)	-12.37 ± 9.83	<0.001	-4.52 ± 7.01	0.0108
	PP (n=18)	-10.94 ± 11.02	0.0011	-4.11 ± 8.04	0.0962
Trait Anger	mITT (n=27)	-9.44 ± 8.28	<0.001	-2.52 ± 6.42	0.0747
	PP (n=18)	-8.50 ± 8.28	0.0010	-1.78 ± 6.32	0.2944
AAQ-II	mITT (n=27)	-13.37 ± 9.69	<0.001	-6.04 ± 6.26	<0.001
	PP (n=18)	-13.56 ± 10.41	<0.001	-6.39 ± 6.38	0.0065
EQ-5D Index	mITT (n=27)	0.14 ± 0.21	<0.001	0.05 ± 0.11	0.0390
	PP (n=18)	0.15 ± 0.23	0.0058	0.06 ± 0.11	0.0741
EQ-5D VAS	mITT (n=27)	18.30 ± 23.41	<0.001	6.07 ± 15.09	0.0747
	PP (n=18)	20.83 ± 22.90	0.0019	10.28 ± 16.22	0.0676

Part B. HB-VAS 15-item Changes (0W→8W and 4W→8W)

No	Symptom (mITT, n=27)	0W→8W Change	p-value (FDR)	4W→8W Change	p-value (FDR)
01	Chest tightness/breathlessness	-16.07 ± 20.17	<0.001	-4.19 ± 16.26	0.2065
02	Rising sensation	-18.96 ± 22.08	<0.001	-6.85 ± 13.88	0.0373

**03**	**Heat sensation (face/chest)**	-24.70 ± 24.69	<0.001	-12.33 ± 21.20	0.0195
**04**	**Lump sensation (throat/epigastrium)**	-25.44 ± 24.34	<0.001	-10.22 ± 21.55	0.0373
**05**	**Feeling of injustice/resentment**	-22.63 ± 25.53	<0.001	-8.37 ± 21.51	0.0715
**06**	**Accumulated anger/rage**	-26.07 ± 25.00	<0.001	-9.67 ± 18.55	0.0373
**07**	**Palpitations**	-25.89 ± 28.78	<0.001	-11.85 ± 23.66	0.0373
**08**	**Sleep disturbance**	-16.67 ± 21.80	<0.001	-2.48 ± 18.04	0.2447
**09**	**Headache/dizziness**	-27.78 ± 29.16	<0.001	-9.85 ± 21.09	0.0373
**10**	**Dry mouth**	-23.37 ± 28.90	<0.001	-10.65 ± 22.29	0.0373
**11**	**Loss of appetite**	-21.96 ± 28.21	<0.001	-7.98 ± 21.26	0.0715
**12**	**Fearfulness/startle response**	-24.67 ± 26.85	<0.001	-9.93 ± 25.54	0.0715
**13**	**Intrusive thoughts**	-21.26 ± 29.68	0.0010	-10.44 ± 16.40	0.0142
**14**	**Frequent sighing**	-28.15 ± 27.18	<0.001	-14.48 ± 19.94	0.0135
**15**	**Deep-seated resentment (Han)**	-24.85 ± 30.47	<0.001	-8.00 ± 21.22	0.0715

Table S10. HB screening status (HBSS ≥ 30) across assessment timepoints (mITTs).

Part A. Screening status at each time point

Timepoint	N assessed	Screen-positive, n (%)	Screen-negative, n (%)
**0W (Baseline)**	28	28 (100.0)	0 (0.0)
**2W**	27	25 (92.6)	2 (7.4)
**4W (Post-treatment)**	28	23 (82.1)	5 (17.9)
**8W (Follow-up)**	27	15 (55.6)	12 (44.4)

Part B. Conversion from baseline (0W) to Week 4

Category	n (%)
**Screen-positive at 0W (N = 28)**	
**→ Converted to negative at 4W**	5 (17.9)
**→ Remained positive at 4W**	23 (82.1)
**Screen-negative at 0W (N = 0)**	
**→ New positive at 4W**	0 (0.0)

Note. McNemar's test not applicable at 0W vs. 4W as all participants were screen-positive at baseline.

Table S11. Mixed model for repeated measures (MMRMs) results for six primary clinical scales (mITT).

Part A: Model fit indices and group variance

Scale	Observations	Log-Likelihood	AIC	BIC	Group Var (SE)
**HB Symptom**	82	-284.28	576.56	586.18	36.90 (2.67)
**BDI-II**	82	-290.53	589.06	598.68	34.29 (2.42)
**STAI-S**	82	-236.56	481.12	490.75	6.86 (1.13)
**STAI-T**	82	-230.50	468.99	478.62	9.53 (1.36)
**STAXI-S**	82	-260.71	529.43	539.05	31.39 (2.84)
**STAXI-T**	82	-246.08	500.16	509.79	21.82 (2.38)

Part B: Fixed effects estimates: coefficients and p-values

Scale	Intercept (p)	Week 4 (p)	Week 8 (p)	Baseline (p)
**HB Symptom**	28.720 (0.001)	-4.419 (0.015)	-10.391 (<0.001)	0.276 (0.110)
**BDI-II**	22.997 (<0.001)	-5.312 (0.009)	-8.752 (<0.001)	0.204 (0.129)
**STAI-S**	32.973 (<0.001)	0.563 (0.595)	-2.494 (0.020)	0.228 (0.130)
**STAI-T**	30.558 (<0.001)	0.263 (0.773)	-1.369 (0.139)	0.259 (0.044)
**STAXI-S**	15.643 (0.002)	-2.971 (0.017)	-7.307 (<0.001)	0.291 (0.084)
**STAXI-T**	15.749 (0.001)	-1.369 (0.185)	-3.934 (<0.001)	0.288 (0.054)

Note. Discrepancies between MMRM fixed-effect estimates and paired-test results (e.g., STAI-T and STAXI-T significant by paired test but not by MMRM at Week 4) reflect differences in model assumptions: paired tests compare observed means at each timepoint without adjusting for baseline or the covariance structure across visits, whereas MMRM estimates marginal change conditional on baseline and accounts for within-subject correlation across all timepoints simultaneously. These two approaches are complementary and not directly comparable.

Table S12. Hwa-byung Symptom Scale exploratory responder analysis (mITT).

Timepoint	Scale	Criterion	N of analyzed	N of responders	Responder rate(%)
Week 4	Hwa-byung Symptom Scale	≥30% decrease from baseline	28	8	28.6
Week 8	Hwa-byung Symptom Scale	≥30% decrease from baseline	27	16	59.3

Note. Responder criterion: ≥ 30% decrease from baseline HBSS. This threshold was not pre-specified and was adopted in the absence of an established MCID for the HBSS; findings are hypothesis-generating only.

Table S13. Exploratory mediation path estimates and bootstrap indirect effect.

Part A. Mediation Path Estimates and Statistical Associations

Path	Description	Estimate (β/ϱ)	Statistic / SD	p-value
**Path a**	ΔAAQ change from baseline	-7.036	SD = 8.87	0.0011†
**Path c**	Total effect (Baseline HBSS → ΔHBSS)	-0.798	β = -0.798	0.0007
**Path b**	ΔAAQ → ΔHBSS (adj. Baseline HBSS)	0.415	β = 0.415	0.0410
**Path c'**	Direct effect (Baseline HBSS → ΔHBSS ∣ ΔAAQ)	-0.595	β = -0.595	0.0105
**Correlation**	Spearman ϱ (ΔAAQ vs. ΔHBSS)	0.532	ϱ = 0.532	0.0035
**Model Fit**	R^2 (Full mediation model)	0.463	-	-

Note. †, Calculated using Wilcoxon signed-rank test (W = 41.5).

Part B. Bootstrap Indirect Effect Analysis

Effect	Mean Estimate	Bootstrap 95% CI (Lower)	Bootstrap 95% CI (Upper)	Significant
Indirect effect	-3.0156	-6.4187	-0.4030	Yes

Note. Bootstrap results based on 5,000 resamples (seed = 42). Significance is determined by the 95% CI excluding zero. All mediation findings are exploratory and should be interpreted with caution given the single-arm pre-post design; causal inference is not warranted.

Part C. Exploratory Logistic Regression for Clinical Outcome

Model	Independent Var.	Odds Ratio	p-value	N
HB Screening Status (4W)	ΔAAQ	1.226	0.0286	28

Note. Model adjusted for baseline HBSS. Outcome defined as screen-positive status at Week 4.

Table S14. Adherence–outcome correlation: Spearman's ϱ between app use days (minimum and maximum) and change in clinical outcomes from baseline to Week 4 (mITT).

Outcome (Δ Baseline to 4W)	Adherence Metric	Spearman's ϱ	p (raw)	p (FDR)
HB Symptom Scale	Min use days (app launch)	0.025	.898	.982
	Max use days (full session)	0.149	.450	.982
Depression (BDI-II)	Min use days (app launch)	-0.087	.661	.982
	Max use days (full session)	0.004	.982	.982
State Anxiety (STAI-S)	Min use days (app launch)	-0.202	.303	.982
	Max use days (full session)	-0.054	.786	.982
Trait Anxiety (STAI-T)	Min use days (app launch)	0.056	.776	.982
	Max use days (full session)	0.196	.318	.982
State Anger (STAXI-S)	Min use days (app launch)	0.059	.767	.982
	Max use days (full session)	0.122	.535	.982
Trait Anger (STAXI-T)	Min use days (app launch)	0.018	.926	.982
	Max use days (full session)	0.103	.603	.982
Acceptance (AAQ-II)	Min use days (app launch)	-0.013	.947	.982
	Max use days (full session)	0.112	.571	.982

Note. Spearman's rank correlation coefficient (ϱ) was utilized to analyze the relationship between adherence and clinical outcomes in the modified intention-to-treat (mITT) population (N = 28). Adherence was operationalized as the number of days with app usage, ranging from 'Minimum' (days with at least one app launch) to 'Maximum' (days with at least one full diaphragmatic breathing session completion). Benjamini-Hochberg FDR correction was applied across all 14 correlation pairs, and no statistically significant associations were observed ($p < .05$). All p-FDR values resulted in approximately .982, indicating that the magnitude of clinical improvement was independent of the frequency of app usage or session completion in this study.
